# Taxonomic features and comparisons of the gut microbiome from two edible fungus-farming termites (*Macrotermes falciger*; *M. natalensis*) harvested in the Vhembe district of Limpopo, South Africa

**DOI:** 10.1186/s12866-019-1540-5

**Published:** 2019-07-17

**Authors:** Stephanie L. Schnorr, Courtney A. Hofman, Shandukani R. Netshifhefhe, Frances D. Duncan, Tanvi P. Honap, Julie Lesnik, Cecil M. Lewis

**Affiliations:** 1 Konrad Lorenz Institute for Evolution and Cognition Research, Klosterneuburg, Austria; 2 Department of Anthropology, University of Oklahoma, Norman, OK USA; 3 Laboratories of Molecular Anthropology and Microbiome Research, University of Oklahoma, Norman, OK USA; 4 Department of Anthropology, University of Nevada, Las Vegas, Las Vegas, NV USA; 5 School of Animal, Plant and Environmental Sciences, University of the Witwatersrand, Johannesburg, South Africa; 6 Gauteng Department of Agriculture and Rural Development, Johannesburg, South Africa; 7 Department of Anthropology, Wayne State University, Detroit, MI USA

**Keywords:** Entomophagy, Termite, Gut microbiome, Human diet, Other faunivory, Human evolution, Macrotermitinae, *Macrotermes*, *Termitomyces*, *Treponema*

## Abstract

**Background:**

Termites are an important food resource for many human populations around the world, and are a good supply of nutrients. The fungus-farming ‘higher’ termite members of Macrotermitinae are also consumed by modern great apes and are implicated as critical dietary resources for early hominins. While the chemical nutritional composition of edible termites is well known, their microbiomes are unexplored in the context of human health. Here we sequenced the V4 region of the 16S rRNA gene of gut microbiota extracted from the whole intestinal tract of two *Macrotermes sp.* soldiers collected from the Limpopo region of South Africa.

**Results:**

Major and minor soldier subcastes of *M. falciger* exhibit consistent differences in taxonomic representation, and are variable in microbial presence and abundance patterns when compared to another edible but less preferred species, *M. natalensis*. Subcaste differences include alternate patterns in sulfate-reducing bacteria and methanogenic Euryarchaeota abundance, and differences in abundance between *Alistipes* and *Ruminococcaceae*. *M. falciger* minor soldiers and *M. natalensis* soldiers have similar microbial profiles, likely from close proximity to the termite worker castes, particularly during foraging and fungus garden cultivation. Compared with previously published termite and cockroach gut microbiome data, the taxonomic representation was generally split between termites that directly digest lignocellulose and humic substrates and those that consume a more distilled form of nutrition as with the omnivorous cockroaches and fungus-farming termites. Lastly, to determine if edible termites may point to a shared reservoir for rare bacterial taxa found in the gut microbiome of humans, we focused on the genus *Treponema*. The majority of *Treponema* sequences from edible termite gut microbiota most closely relate to species recovered from other termites or from environmental samples, except for one novel OTU strain, which clustered separately with *Treponema* found in hunter-gatherer human groups.

**Conclusions:**

*Macrotermes* consumed by humans display special gut microbial arrangements that are atypical for a lignocellulose digesting invertebrate, but are instead suited to the simplified nutrition in the fungus-farmer diet. Our work brings to light the particular termite microbiome features that should be explored further as avenues in human health, agricultural sustainability, and evolutionary research.

**Electronic supplementary material:**

The online version of this article (10.1186/s12866-019-1540-5) contains supplementary material, which is available to authorized users.

## Background

Insects have long been an important food resource for primates, likely extending back to the origins of the order in the Paleocene [1, 2]. For modern human populations, entomophagy (consumption of insects) serves both biological and cultural purposes as a nutritional support and as an edifice of group identity in food sovereignty [3]. Termites in particular are an important dietary supplement for great apes and humans [4, 5], and they have been postulated as a bridge resource for early hominins transitioning from dense forest to savannah-mosaic environments [6]. This is suggested in part by observations that other great apes (mainly chimpanzees and bonobos) forage for termites, and because termites in savanna-woodlands biomes, particularly the fungus-farmers of *Macrotermes*, present a mixed C3/C4 stable carbon isotope composition, with ^13^C enrichment in the non-reproductive castes, that is similar to Australopithecine isotopic profiles [7, 8].

One relevant distinction between entomophagy and traditional faunivory is that insects are consumed in their entirety unlike other animal foods, which tend to be separated and consumed on a tissue-specific basis [9, 10]. Therefore, insects provide the consumer with some unique and possibly rare nutritional components that are less well understood in terms of their impact on consumer physiology. Such components include chitin (a carbohydrate polymer of N-acetylglucosamine that forms arthropod exoskeletons), exotic hydrocarbons from venoms, toxins, or cuticular signaling molecules [11–13], and other somatic tissues, particularly the digestive tract and its contents. In consuming the digestive tract, one also acquires the enteric microbial environment (the gut microbiome), which comprises microbial cells, genetic information, chemicals, and food residues that together potentially behave as both a prebiotic and probiotic to the consumer. A limitation however is that dietary elements reaching the colon have already undergone digestion in the upper gastro-intestinal tract, making it difficult to estimate whether microbiota may survive this journey. Humans possess chitinase enzymes [14, 15] that can break down exoskeleton material, making the interior contents highly susceptible to enzymatic digestion. However, prior research shows that fecal contents of great apes contain macro- and microscopic remains of exoskeletons [16], a counter to suggestions that insect soma may be primarily digested in the proximal gut. We postulate that molecular substrates in the form of genetic material, proteins, and bacterial cell membranes have the potential to influence endemic human microbial communities residing in the colon. Therefore, one hypothetical implication is that insects are a whole-food microbiome “seed” in a single bite. In this study, we characterize what microbes such a bite could entail.

This study is the first to sequence the gut microbiota from termites directly observed to be consumed by humans. For two edible *Macrotermes* species, we characterize the microbiome of individual edible termites within the soldier caste (subcastes), between these subcastes, and finally between species, revealing that each such bite may vary substantially. We determine if such edible termites may serve as reservoirs, or point to shared environmental sources, for a spirochete found to be common to the gut microbiome of traditional peoples. The microbiome variation observed leads us to new hypotheses regarding termite ecology.

### Termite phylogeny and ecology of fungus-farmers

According to estimates of mitochondrial molecular divergence, all extant termites belong to the infraorder Isoptera within the order Blattodea [17]. Research on Isoptera adds another layer of intrigue to the microbiome implications of entomophagy. Termites are primary degraders of plant material and detritus during decomposition, including wood, grass, soil, dung, and leaves. However, termites are unable to directly digest the materials they acquire from the environment; hence they have evolved uncanny interdependent symbioses with enteric microorganisms and domesticated fungi. In these relationships, the burden of enzyme production for cellulose and xylan hydrolysis falls upon the symbiotic microorganisms and fungus, and the termites are nutritionally supported by symbiont metabolites of acetate, carbohydrates, and amino acids [18]. The particular symbiotic community varies depending on the termite taxon. The ‘lower’ termites are primarily wood-feeders dispersed across several taxonomic families that rely on cellulose-fermenting protozoa or other flagellate microbiota to breakdown lignocellulose [19]. The ‘higher’ termites are members of Termitidae with diverse diets and harbor only bacteria and archaea in their gut ecosystem, which for the wood-feeders is usually dominated by the genus *Treponema* [18–20]. The deviation from this pattern occurs with members of the subfamily Macrotermitinae in the Termitidae family, who cultivate gardens of a domesticated *Termitomyces* fungus inside of the nest mound [21] and feed from the fungus comb and nitrogenous ‘mycotêtes’ conidia. Gut microbiome rearrangements from those of wood-feeding and soil-feeding termites have been observed for Macrotermitinae such that functional complementarity arises between termite and fungal genomes to support termite nutritional acquisition [22]. Previous studies on the gut microbiome profile of fungus-farming termites have looked at various species in the Macrotermitinae clade, including *Macrotermes natalensis*, *M. gilvus*, *M. subhyalinus*, *M. annandalei*, *M. michaelseni*, *Microtermes sp*., *Odontotermes sp*., *Ancistrotermes sp*., and *Pseudacanthotermes sp*. [22–29], however, most of the in-depth compositional profiles are derived from the worker caste, and to our knowledge, no data exist for the primary species, *Macrotermes falciger*, that is targeted by hominin consumers. Based on these prior assessments, the fungus-farming termite microbiome profile is markedly different from that of soil-, litter-, and wood-feeding termites. Prominent features of the fungus-farmer gut microbiome include metagenomic specialization for oligosaccharide metabolism rather than for complex polysaccharide degradation [22], a reduction in *Treponema* relative to non-fungus-farming termites, and broad taxonomic similarity to the noneusocial insect sister clade of cockroaches dominated by *Ruminococcaceae*, *Alistipes*, *Clostridium*, and *Lachnospiraceae*.

Complex nutritional cycling and labor coordination of the Macrotermitinae affords an opportunity for unique microbiome composition even among individuals within a termite colony. The Macrotermitinae monophyletic clade of 11 genera uniquely maintain an obligate symbiosis with the termite-associated fungal genus, *Termitomyces*. Neither fungus nor Macrotermitinae can survive independent of the other [21, 30]. The partnership manifests in a cycle that involves initial inoculation of the termite brood with the *Termitomyces* fungal spores by the founding queen [31], and then cultivation and maintenance of the fungal gardens by the sterile worker caste. As the workers mature, they instate a complex division of labor, or polyethism, from young to old workers. The old workers bring foraged plant materials inside the mound, undigested, and the young workers ingest and inoculate these materials with the *Termitomyces* spores via rapid passage of the plant material through their digestive tract. The young workers then defecate the inoculated plant material as a sponge structure at “garden” sites deep inside the mound that eventually develop into mature fungus comb. Once mature, the *Termitomyces* produces small white conidia nodules rich in nitrogen that the termites consume [24]. The entire process constitutes a two-stage digestion for the termite colony: the first to inoculate organic matter with the fungus, and the second to actually consume the cultivated fungal growths for nutritional benefit. Morphotype differentiation (e.g. major and minor subcastes) in feeding behavior for both workers and soldiers is apparent. Young minor and major workers as well as minor soldiers consume the nitrogen rich conidia in order to develop the somatic tissues necessary to carry out their essential duties as mature colony members. Worker duties entail nourishing the larvae, the soldiers, and the reproductive castes via trophallaxis, while the soldiers must develop large mandibular structures for defense of the nest. Older workers feed exclusively from the old fungal comb, and in turn feed the major soldiers [24]. The elaborate nutritional cycling and labor coordination of the Macrotermitinae is astounding, and thus warrants careful study of morphotype or sub-caste variation in physiology and microbiome composition.

### Roles of termites in human and primate diets

Termite foraging has been observed by all of the extant great apes and modern humans have been harvesting termites for millennia [32]. While chimpanzees strongly prefer soldiers of *Macrotermes*, gorillas are known to target the workers of *Cubitermes* [5]. These preferences may reflect overall differences in diet, whereby frugivorous chimpanzees target protein-rich termites and folivorous gorillas target micronutrient-rich termites. Human preferences are more variable; there are about 30 species from 13 genera of termites recorded as food around the world. Of these, eleven species belong to the genus *Macrotermes* [33]. People often target the seasonally available flying reproductives, or alates, but also collect soldiers year-round. Like chimpanzees, people can take advantage of the biting mandibles of *Macrotermes* soldiers by using a tool made of grass or other vegetation [34]. While chimpanzees thread a single stem into a tunnel in the termite nest, people increase their foraging efficiency by excavating a large opening into the nest and dipping in a rudimentary broom. The soldier termites attack these tool “invaders” and are easily extracted from the nest.

Termite consumption can supplement macronutrients such as fat and protein and also fulfill certain micronutrient daily requirements [5, 35–37]. These benefits make termites a compelling food option in reconstructions of early hominin diet [38]. Today termite mounds from multiple *Macrotermes* species litter the East African Rift Valley and extend all the way down to South Africa into the Sterkfontein Valley [6, 39] and there is good indication that *Macrotermes* would have been available in these areas over the course of hominin evolution [40, 41]. Fungus farming termites from the *Macrotermes* genus are preferentially exploited by humans and chimpanzees due to their large size, consistent occupancy of the nest, their easy access within the nest, and because of their high protein content relative to other termites [5]. Each mound houses a large number of active sterile soldier castes for a windfall of easy animal protein, while the winged alates are rich in fatty acids and a good source of calories [38]. One plausible role of termite consumption is that the mineral and humic contents of their guts function as a form of geophagy to help absorb toxins, prevent diarrhea, or remove enteric parasites [5, 42, 43]. Curiously, local women from the Vhembe District in the province of Limpopo, South Africa who regularly harvest termites [44] conveyed, though anecdotally, to one of the authors (Netshifhefhe) that eating soldier termites eases digestion and claimed that they are very helpful to those with constipation problems. Far-reaching notions aside, there are many reasons to pursue investigations that focus on the interaction between the termite and human microbiomes; however, work to date on the gut microbiome of fungus-farming termites has prioritized the worker caste. Few studies exist that include genomic analysis of the soldier-caste microbiome [22, 24, 45] and even these have failed to differentiate between sub-caste morphotypes of major and minor soldiers.

In the present study we characterized the bacterial taxonomic profile of soldier castes from two wild *Macrotermes* species acquired from South Africa with the aim of assigning concrete biological features to the visibly distinctive soldier sub-castes. Since humans and great apes exclusively consume the soldier caste, and particularly the major soldiers where they occur, of *Macrotermes* species, it is important to know whether distinguishing gut microbiome features of these morphotypes exist. Considered in this study is that edible termites may provide clues to an inoculation source of *Treponema sp.* that are members of the gut microbiome observed in traditional, non-industrialized peoples. This genus has been of strong interest in human microbiome research because it appears to be a member of the primate/mammal gut microbiome that was extirpated by a yet unknown process attributed to industrialization [46–52]. We also maintain an anthropological perspective on how consumption of soldier-caste termites may impact human physiology, particularly as it relates to digestive health, and we attend to the broader implications of the possibility that ontogenetic dietary adaptations are facilitated by the gut microbiota throughout human evolution.

## Results

A total of 85 dissections of termite specimens from five different termite mounds (Vhembe 1, Vhembe 4, Vhembe 6, Vhembe 7, and Vhembe 8) resulted in 67 usable termite gut microbiome samples that were prepared and sequenced. Most samples are individual guts from single termites, but a total of 4 samples come from extractions done on three pooled termite guts for each sample, a total of 10 samples come from extractions done on fractioned (0.5) termite guts, and a total of 2 samples come from extractions done on five pooled fractioned guts. A full summary of the sample origins, metadata, and sequence data information is provided in Additional file 1: Table S1. Targeted amplifications of the V4 hypervariable region of the 16S rRNA bacterial/archaeal gene were sequenced on Illumina platforms (MiSeq and NextSeq) across three different runs (Run1, Run2, and Run3), resulting in an average of 23048 (±11147 SD), 18332 (±12259 SD), 90422 (±59916 SD) sequences per sample (not including positive or negative controls) for each run respectively. All sample sequence data were combined for denovo clustering to derive operational taxonomic units (OTUs) and the final OTU table of the combined runs was rarefied to a depth of 8000 for the main analyses.

### Gut ecology validation

In order to learn about the range of variation of the termite gut microbiome for the *M. falciger* and *M. natalensis* species, we were ideally interested in characterizing the gut microbiome at the level of individual termites. To do this, we needed to determine whether a single termite gut contained enough biological material to reliably reconstruct the gut microbial ecology, since nearly all prior work and protocols homogenized pooled guts into a single aliquot for extraction [22, 23, 53, 54]. We therefore conducted in-depth analysis on a subset of the full dataset that derived from a batch run prepared using single, pooled, and fractioned termite guts. These samples were also used to assess whether differences in gut mass, pooling, DNA yield, or sequencing depth would lead to skewed representation of the termite gut ecology in diversity or taxonomy.

Sequencing success was variable, with nine samples yielding < 5000 final filtered FASTA reads, and two samples yielding < 3000 filtered FASTA reads. Therefore, we first looked at whether sequencing depth significantly affected the OTU profiles. Procrustes rotation of the Bray-Curtis dissimilarity matrix for OTU tables rarefied to 1000 and 14000 reads respectively shows significant non-random conformity between matrices, with a 0.998 permutation symmetry correlation score based on a Monte-Carlo resampling process using the function “protest” in the package {vegan} in R (m2 = 0.0043, *p* = 0.001) (Additional file 4: Figure S2A). The Procrustes analysis indicates that taxonomic distribution is not meaningfully altered from the lowest to the highest rarefaction depth. A final rarefaction depth of 3000 was chosen as the highest depth that omits the fewest low-abundance samples. Again, using Procrustes rotations of the Bray-Curtis distance-matrices, the sample matrix of the single-gut extractions was compared to each fractioned and pooled sample matrices (Additional file 4: Figure S2B), resulting in good correlation between the target and rotated datasets (single vs fraction: correlation = 0.92, m2 = 0.144, *p* = 0.007; single vs pool: correlation = 0.89, m2 = 0.203, *p* = 0.25). Using the OTU table, both an ordination using Bray-Curtis dissimilarity and a heatmap show that samples intersperse randomly based on extraction method, and still maintain biological groupings based on soldier type - major or minor (Additional file 4: Figure S2C). Comparisons of the OTU abundance table by extraction method, using permutational multivariate analysis of variance (PERMANOVA) within soldier caste type (using strata in the function “adonis” in {vegan}), confirms that the microbial profile cannot be differentiated based on the use of single, fractioned, or pooled starting gut material (PERMANOVA pseudo-F ratios, *R*^*2*^ = 0.08, *p* > 0.5). These results demonstrate that single guts faithfully represented the full termite gut ecology as near as can be approximated using the V4 bacterial/archaeal 515F/806R primer set.

Correlations testing was conducted to look for any biased associations due to gut mass (mg), extracted DNA concentrations, and cycle threshold (Cq value) on alpha-diversity as well as taxonomic abundance. Pairwise Pearson correlation indicates that these individual properties of each sample do not impact overall diversity capture and taxonomic results (Additional file 5: Figure S3A). This is important because it suggests that the data are not biased by procedurally separate components of data procurement. Instead, physical attributes (mass), extraction yields (DNA concentration), and amplification cycle thresholds co-correlate, as do the alpha diversity metrics (OTU count and phylogenetic diversity), which is to be expected. Finally, Spearman cross correlation between the taxonomic abundance and the metadata (from above) indicates whether taxonomic distributions are impacted by extraction variables. Aside from positive correlations between alpha-diversity and several taxa (to be expected, since higher diversity resolves more taxa), no physical parameter significantly correlates with any taxon (Additional file 2: Table S2). Neither DNA concentration from extractions nor Cq-value from qPCR significantly correlate with taxonomic abundance, indicating that DNA yield and amplification have not biased the reconstruction of the microbial membership.

### Positive controls

In order to understand the source and extent of batch effects on sample sets that were extracted, amplified, and sequenced in different groups, we were able to look to the profile of the positive control samples. Theses samples come from human fecal DNA that was extracted using the MoBio PowerSoil kit following manufacturer recommendations. As already-extracted samples, the controls were thus used alongside each of the termite sample batches (Run1, Run2, and Run3) beginning from PCR amplification. The positive controls all amplified successfully and achieved an average of 43,424 merged FASTQ reads (min = 20,745, max = 55,250; Additional file 1: Table S1). Visualized with the combined datasets from all batch runs, the positive controls clustered most closely together in ordination plots using unweighted and weighted UniFrac distance, as well as Bray-Curtis dissimilarity (Additional file 6: Figure S4A). Hierarchical ward clustering of the unweighted UniFrac matrix also splits the positive controls away from the rest of the sample set at the highest branch (Additional file 6: Figure S4B). Permutational multivariate analysis of variance of the positive controls indicates no difference (PERMANOVA pseudo F-ratios, *R*^*2*^ = 1, *p* = 1), and a two-sided Kolmogorov-Smirnov test confirms the null hypothesis that the positive control samples were drawn from the same distribution (D = 0.0126, *p* = 1). These findings are important for supporting the inference that all post-extraction handling and data curation of the three batch runs did not perceivably bias the resulting microbial profiles of the samples. Thus, we cannot exclude the possibility that batch bias did occur as a result of DNA extraction, however batch differences are potentially actual biological differences between different mounds.

### Termite data results

#### Alpha-diversity

**Fig. 1:**
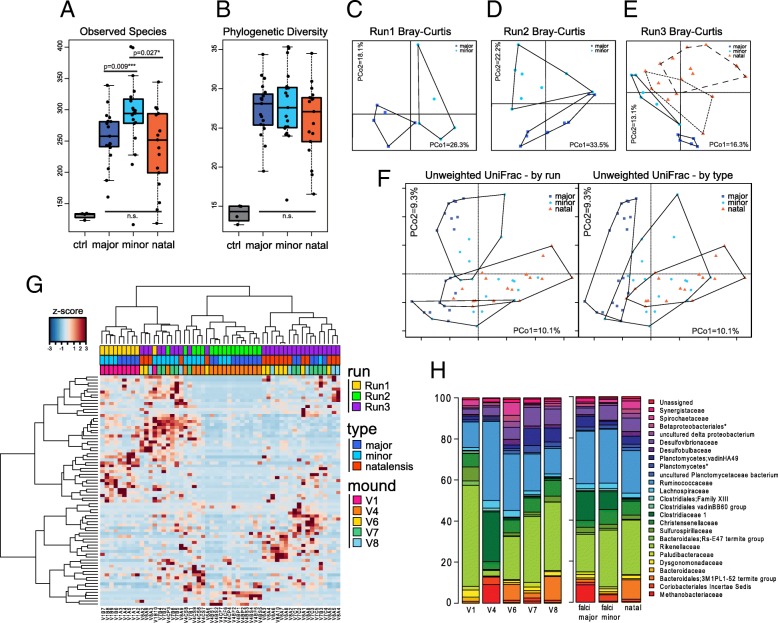
Alpha and beta diversity comparisons across runs and soldier specimen types show caste-based distinctions. **a, b** Boxplots of Observed Species and Faith’s Phylogenetic Diversity metrics for major and minor *M. falciger* soldiers (dark and light blue) and for *M. natalensis* soldiers (orange), with significances of between group comparisons shown. **c-e** Ordination of OTU-level relative abundance using Bray-Curtis dissimilarity shows caste (major/minor) and species (*M. falciger*/*M.natalensis*) separation by run (top plots) and outlined by caste (for *M. facliger* solid lines) and by mound (for *M. natalensis* dashed lines), and **f** unweighted UniFrac distance on all samples combined across runs shows that samples stratify by run on PCo2 and by type on PCo1. **g** Heatplot of OTUs filtered for taxa at ≥0.1% abundance in at least 10% of samples shows z-score levels of the OTU relative abundance clustered by Ward’s method for both OTUs (rows) and samples (columns). Samples are colored along the top row by run, type, and by mound. **h** Barplots of summarized family-level taxa at 1% abundance in at least 20% of samples averaged by mound and by termite species/caste

Microbiome alpha-diversity was analyzed using Observed Species and Faith’s Phylogenetic Diversity metrics on the rarefied OTU table. Variation exists only in Observed Species (i.e. OTUs) between *M. falciger* soldier castes and the minor caste as well as between the *M. natalensis* species and minor caste of *M. falciger* (Wilcoxon, *p* = 0.009 and *p* = 0.027; Fig. 1a). Variation between all soldiers of each termite species is not significant. Curiously, *M. falciger* minor soldiers have the highest overall diversity.

#### Beta-diversity

Ordination of each batch run from the rarefied OTU table using the Bray-Curtis dissimilarity matrix shows a consistent pattern of significant clustering by caste within *M. falciger*, and within the Run3 batch run a clear separation between *M. falciger* majors and minors and *M. natalensis* (PERMANOVA pseudo F-ratios: Run1 *R*^*2*^ = 0.22, *p* = 0.011; Run2 *R*^*2*^ = 0.18, *p* = 0.015; Run3 *R*^*2*^ = 0.30, *p* = 0.005; and Run3 species *R*^*2*^ = 0.10, *p* < 0.001 respectively; Fig. 1c-e). The separation of *M. natalensis* samples by-mound is apparent within Run3 (Fig. 1e dashed polygons) and is modestly significant (PERMANOVA pseudo F-ratios: *R*^*2*^ = 0.12, *p* = 0.014), demonstrating by-mound variation that is not impinged by batch effects. Using the combined run data, ordination using the unweighted UniFrac distance matrix shows that samples segregate by type (major, minor, *M. natalensis*) along PCo1, and by run along PCo2. The combined data indicate that meaningful biological variation exists between castes and species that is robust to batch effects and merits further investigation (PERMANOVA pseudo F-ratios, type *R*^*2*^ = 0.12 and batch *R*^*2*^ = 0.13, both *p* < 0.001; Fig. 1f). There is also good evidence for biological separation by mound – the *M. falciger* mounds: Vhembe 1 and Vhembe 4 for Run1 and Run2 respectively; Run3 consisted of two *M. natalensis* mounds: Vhembe 6 and Vhembe 8, and one *M. falciger* mound, Vhembe 7. This is supported by the ordination plots and the distribution of samples in a heatplot (Fig. 1g) of the rarefied OTU table, filtered for OTUs at 0.1% minimum relative abundance in at least 30% of samples, in which Run1 (yellow) and Run2 (green) form distinct hierarchical clusters, and Run3 (purple) splits largely in accordance with mound or caste membership.

#### Indicator species

**Fig. 2:**
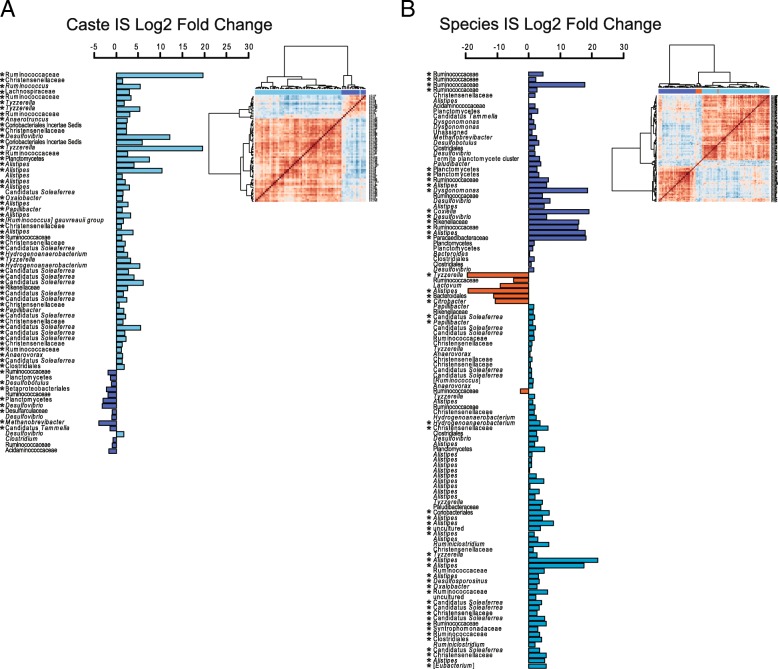
Indicator species OTUs shows distinct taxonomic abundance differences. Indicator species OTUs are ordered by hierarchical clustering of Kendall correlations on relative abundance (inset heatplots) and plotted along an axis of log2 fold change. OTU taxonomic assignments are shown at the lowest level resolved, and asterisked taxa indicate FDR corrected significant values based on Wilcoxon test of abundance. Indicator species comparisons were done between **a** soldier castes of *M. falciger* across all runs, and **b** between termite species of *M. falciger* and *M. natalensis* within Run3

In order to understand the taxonomic differences between major and minor soldiers of *M. falciger* as well as between the termite species *M. falciger* and *M. natalensis*, we employed an indicator species (IS) analysis alongside significance testing on the rarefied OTU table, which accounts for frequency as well as abundance of microbial taxa within defined groups. After removing indicator OTUs that were also IS of batch-run differentiation, a total of 68 OTUs remained with an IS score > 0.6 that differentiated major from minor soldiers of *M. falciger* (Fig. 2a). Hierarchical clustering shows that IS OTUs belonging to minor solders largely co-associate, to the exclusion of IS OTUs belonging to major soldiers (Fig. 2a inset heatmap). Taxonomic assignments for IS OTUs are summarized at the lowest level distinguished by alignment to the SILVA 16S database [55], but due to low resolution, many OTUs are known only at the family level and/or are largely redundant for major and minor soldiers. Thus, while an array of OTUs distinguish *M. falciger* soldier castes, their taxonomic assignments are mainly unresolved at the genus or strain level (see Additional file 7: Figure S5 for relative abundance taxonomic summaries). The few taxa that are uniquely distinctive for major soldiers include *Desulfobotulus*, *Methanobrevibacter,* and Candidatus *Tammella*, while minor soldiers are uniquely distinguished by Candidatus *Soleaferrea*, *Tyzzerella*, *Lachnospiraceae*, *Anaerotruncus*, *Alistipes*, *Papillibacter*, *Christensenellaceae*, *Anaerovorax*, and *Oxalobacter*. The same IS strategy was employed in order to find taxa distinguishing *M. natalensis* from *M. falciger* soldiers, but was calculated only within the batch Run3, the only batch to include *M. natalensis* samples, so as to eliminate batch bias. A total of 113 OTUs received IS scores > 0.6 for *M. natalensis* or *M. falciger* majors and minors (Fig. 2b). Again, IS OTUs show strong within-group association in three distinct hierarchical clusters, however taxonomic resolution is again limited, and of the seven IS OTUs associated to *M. natalensis*, two are unique for this termite species: *Lactovum* and *Citrobacter*. In sum, the IS analysis capably extracts the distinguishing OTUs for each termite group, allowing us to see that abundant biological variation exists among these ecologies.

#### Co-abundance groups

**Fig. 3:**
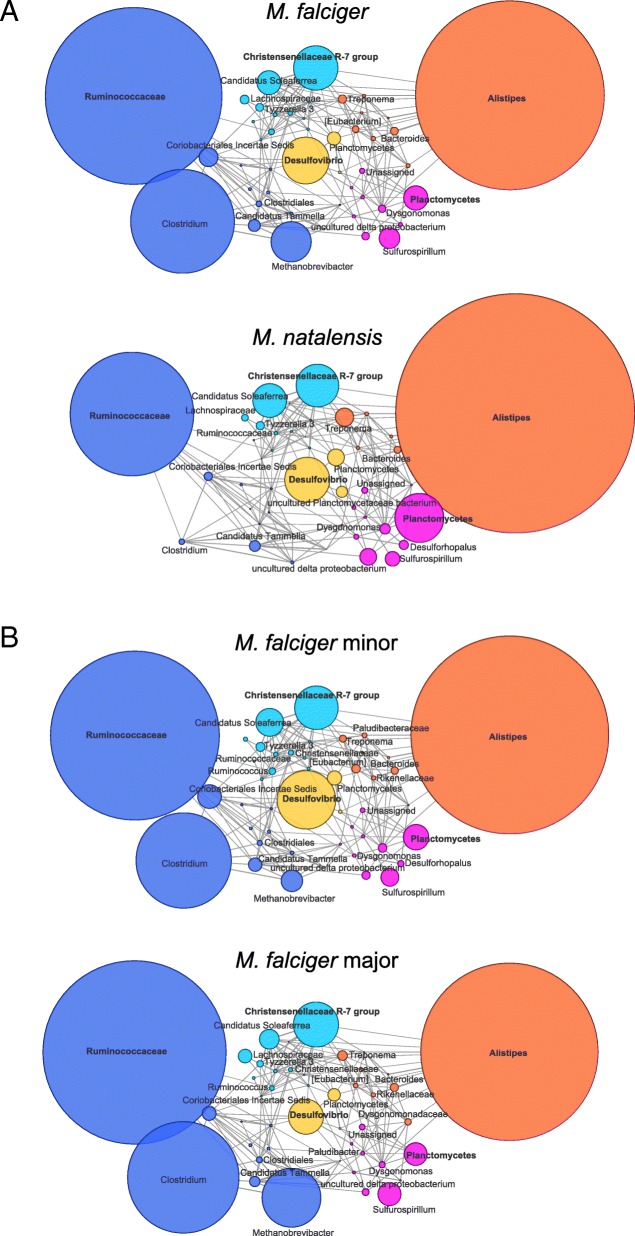
Co-abundance group networks show broad taxonomic fingerprint of each caste and species. Network plots of co-abundance groups are shown by termite species (**a**) and by soldier caste (**b**) within *M. falciger* (bottom two rows). Nodes are colored by co-abundance group: *Ruminococcaceae* (dark blue), *Christensenellaceae* R-7 group (light blue), *Planctomycetes* (pink), *Alistipes* (orange), and *Desulfovibrio* (yellow). Node sizes are proportional to taxonomic abundance and edge widths to correlation coefficient

Since taxonomic resolution is low, a common challenge for novel samples, we determined microbial co-abundance groups (CAGs) and constructed correlation network plots in order to visualize and compare the microbial community organization of these termite microbiomes. After significance testing, five CAGs were found based on hierarchical clustering of positive significant Kendall rank correlations between taxa, and are named for the most abundant taxon within each group: *Ruminococcaceae* (dark blue), *Christensenellaceae* R-7 group (light blue), Planctomycetes (pink), *Alistipes* (orange), and *Desulfovibrio* (yellow) (Additional file 8: Figure S6)*.* The width of the network edges correspond to the correlation coefficient, and the size of the nodes reflect the abundance of each taxon averaged within each termite sample group of interest (Fig. 3). Overall, the network plots are quite similar, however there are characteristic features that differentiate between termite species and between soldier castes. The *M. natalensis* termites are relatively more enriched in the *Alistipes* and Planctomycetes CAGs (orange and pink respectively) than *M. falciger*, which are relatively more enriched in the *Ruminococcaceae* CAG (dark blue) owing to high abundance of *Clostridium* and *Methanobrevibacter* taxa. Differences between major and minor soldiers of *M. falciger* are less pronounced, with majors enriched in the *Ruminococcaceae* CAG (dark blue) relative to minors, which are enriched in the *Alistipes* CAG (orange) and *Desulfovibrio* CAG (yellow). Importantly, *M. falciger* majors are greatly enriched in *Methanobrevibacter,* which is an archaeon member of Euryarchaeota and important for its role as a metabolic end-products scavenger, converting excess hydrogen into methane and preventing over-acidification of the environment that would inhibit primary anaerobic fermentation [56, 57]. *Methanovbrevibacter* is an important member of many complex animal-associated microbial communities as a secondary metabolizer [58], and its presence in the *Macrotermes* gut community is understood to be the source of abundant methane gas produced by fungus-farming termites [54, 59]. Our findings that, unlike *M. falciger* major soldiers, the *M. falciger* minor soldiers and *M. natalensis* soldiers do *not* harbor high relative abundance of methanogens suggests that food substrate access varies within and among fungus-farming soldier castes and species [22, 59]. Therefore, a more nuanced consideration of caste biological and behavioral differences may yield new strategic approaches to *Macrotermes* ecology and agro-economy. In sum, it appears that *M. falciger* major and minor soldiers vary by abundance of *Alistipes* and taxa implicated in secondary metabolism. Additionally, minor soldiers and *M. natalensis* soldiers share a greater reliance on *Alistipes* and the sulfate reducing members of *Desulfovibrio*.

#### Reference sequence meta-analysis

**Fig. 4:**
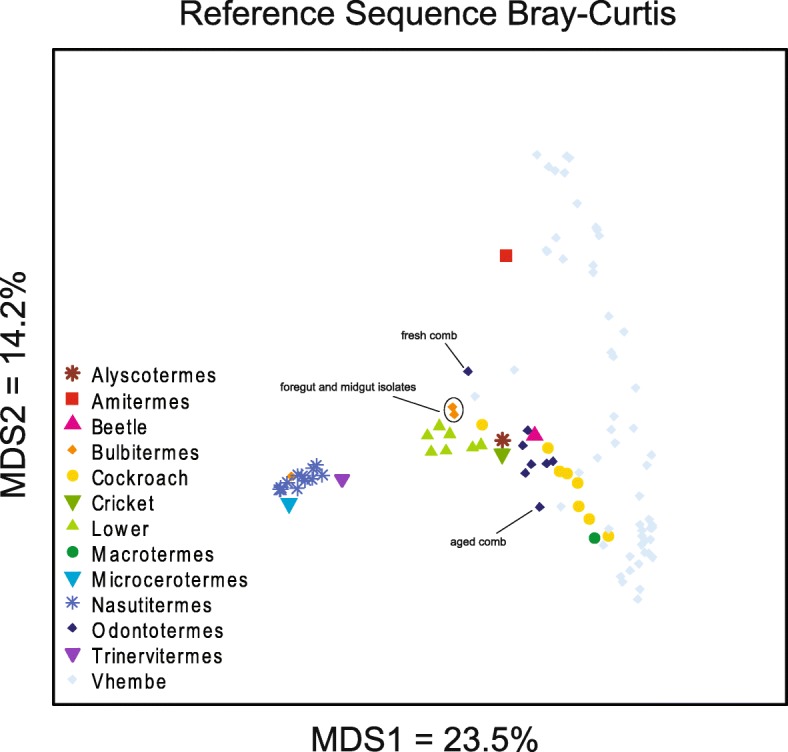
Termite gut microbiome reference sequences vary by host type along PCo1 of Bray-Curtis dissimilarity ordination. Ordination of external meta-taxonomic microbiome data for several termite species alongside the current study data corroborates previous findings that feeding ecology explains correspondence between termite microbiome profiles. Wood-feeding and grass-feeding termites cluster on the left with a gradual shift towards the right with humus and soil feeding lower termites that culminates in a large right-hand cluster of fungus-farmers and omnivores. The latter cluster also spans PCo2, exemplifying the greater microbiome variation of generalist feeders. Foregut/midgut isolates from *Bulbitermes* as well as fungus comb samples from an *Odontotermes* nest are indicated on the plot

**Fig. 5:**
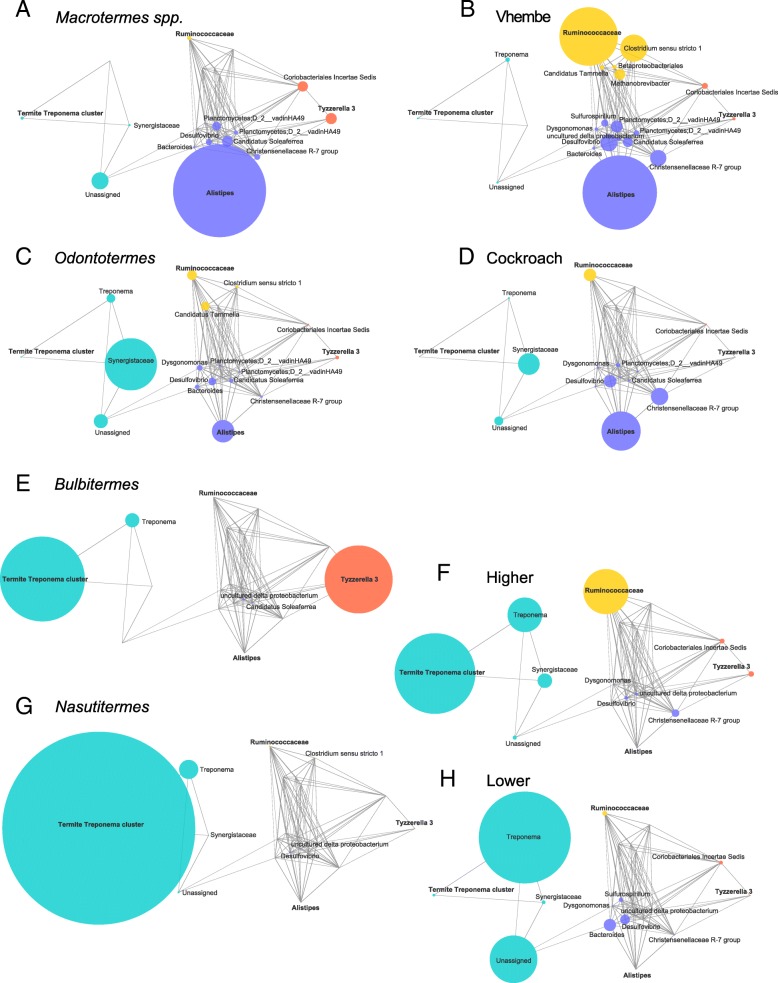
Reference sequence and Vhembe co-abundance networks support stratification of termite microbiomes by lignocellulose degraders versus generalist feeders. Taxonomic abundance in three major clusters, *Ruminococcaceae* (yellow), *Alistipes* (blue), *Tyzzerella* 3 (orange), and *Termite Treponema* cluster (green), shows consistent patterns among the fungus famer and cockroach hosts (**a-d**) that differentiate them from the other wood/grass/soil-feeding termites (**e-h**). The *Alistipes* cluster is best represented with the fungus farmer and cockroach samples, and form a heavily associated network of connected nodes. The *Termite Treponema* cluster CAG is best represented among wood/grass/soil-feeding specimens, which dominates the abundance of most other taxa

In order to compare the ecology we profiled in the Vhembe termites with previous data generated for termite gut microbiota, we acquired 16S rRNA gene sequence data of termite gut microbiota on the NCBI short read archive (SRA) and the literature, resulting in 45 useable external samples sourced across four different studies ([23, 45, 60]; NCBI BioProject PRJNA315033). These studies used variously different gut sections or homogenates of the termite specimens sampled, which reduces the strength of definitive comparisons among gut microbial ecologies for these data. However, these comparisons are valid to the extent that the majority of samples derive from gut homogenates or luminal fluid from the hindgut, which makes up the largest section of the termite gut. See descriptions in Additional file 1: Table S1 for information on particular samples. Ordination of the Bray-Curtis dissimilarity matrix of the genus level taxonomy summary table shows a split along PCo1 in what appears to be a gradient of feeding behavior across termite taxa, with an NMDS plot showing a similar clustering order array (Fig. 4 and Additional file 9: Figure S7). Wood-feeding and litter-feeding taxa such as *Nasutitermes*, *Bulbitermes*, and *Microcerotermes* form a tight cluster with *Trinervitermes* and other lower termite taxa that segregate on the left-hand side. Along the right-hand side is a large cluster spanning PCo2 that includes the Vhembe termite specimens and interspersing specimens belonging to Termitidae family (*Macrotermes sp.* and *Odontotermes*) as well as various species of cockroach. Co-abundance groups were again helpful for consolidating and identifying characteristic traits for each of the different microbiomes. This time, four CAGs were resolved based on significance testing of the hierarchical clustering of the Kendall correlation matrix, and named for the most abundant taxa within each group: *Ruminococcaceae* (yellow), *Tyzzerella* 3 (orange), *Alistipes* (blue), and Termite *Treponema* cluster (green). Network plots showing only the positive significant correlations between taxa were created for each termite taxonomic group, plus the cockroach, beetle, and cricket specimens (Fig. 5 and Additional file 10: Figure S8), with nodes representing the mean relative abundance of microbial taxa. One prominent segregation is that termite taxa are either enriched in *Treponema* or not, and this feature dichotomy corresponds to the stratification seen in the ordination plot. Interestingly, the Vhembe termites sequenced for this study, the *Macrotermitinae* (fungus farming subfamily) taxa, and various cockroach species uniquely share a set of features that includes relative enrichment in the *Alistipes* CAG and depletion in *Treponema* genera or the *Treponema* CAG overall (Fig. 5), supporting observations of the close association between termite evolutionary history, feeding ecology, and microbiome structure [23]. By contrast, the individual wood/grass/litter/humus-feeding termite taxa belonging to the “lower” and “higher” termite groups as well as *Bulbitermes* and *Nasutitermes* genera (both members of Nasutitermitinae subfamily) are predominantly enriched in the *Treponema* CAG, which in some cases comprises over 50% of total taxonomic abundance (Additional file 7: Figure S5B). The pattern of CAG enrichment for two outgroup specimens - scarab beetle larvae (*Pachnoda ephippiata*) and a common field cricket (*Gryllus assimilis*) - bear little resemblance to any of the termite or cockroach CAGs (Additional file 11: Figure S9). However, individual enrichment in the *Alistipes* CAG and the *Ruminococcaceae* and *Tyzzerella* taxa is shared with *Macrotermes spp.*, Vhembe, *Odontotermes*, Cockroaches, and Higher termites. Overall, these findings demonstrate that while certain gut microbiome features of termites are linked to a phylogenetic pattern of microbiome membership, feeding ecology best explains microbial assimilation patterns within Blattodea.

#### Spirochaetes phylogenetic relationships

**Fig. 6:**
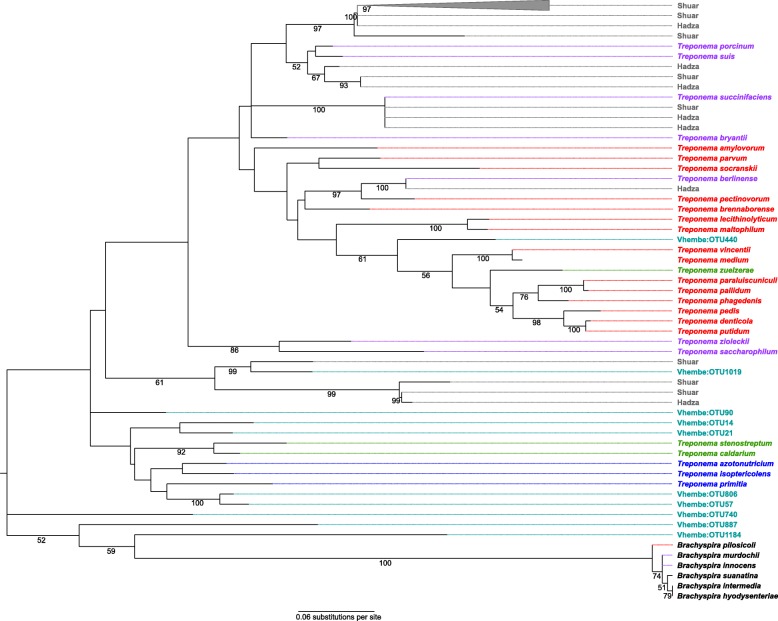
Maximum likelihood tree of Spirochaetes sequences. Spirochaetes OTUs from the Vhembe termite gut microbiome data cluster with other representative Spirochaetes and *Treponema* sequences from environmental and termite sources. Spirochaetes OTUs from human (Hadza and Shuar) gut microbiome data form a subcluster with non-pathogenic *Treponema* isolated from swine separate from a larger cluster of pathogenic *Treponema* pathogens. Taxa are colored as follows: red - pathogenic *Treponema*; purple - non-pathogenic *Treponema*; green - environmental *Treponema*; blue - *Treponema* species associated with termite gut; gray - Spirochaetes OTUs derived from human (Hadza and Shuar) gut microbiomes; turquoise - Spirochaetes OTUs derived from Vhembe termite gut microbiomes (this study)

Exploration of the human gut microbiome across a variety of populations has revealed significant differences in the microbial community membership between small-scale traditional subsistence populations and post-industrial westernized populations [46–48, 50–52, 61, 62]. This includes depletion of certain extirpated bacteria [63] with particular attention paid to the curious presence of Spirochaetes phylum, namely non-pathogenic members of *Treponema* [49], in non-western human groups. Since termites famously exploit the xylan degrading abilities of *Treponema* [64] and the source of human gut treponemes is yet unknown, it was opportune to look for associations between human gut treponemes and the Spirochaetes OTUs found within our Vhembe termite dataset, especially since these termites are regularly consumed by humans. Reference *Treponema* 16S rRNA gene sequence data were downloaded from NCBI, including pathogenic and non-pathogenic strains, as well as non-treponeme members of Spirochaetes (see Methods for reference data curation). Spirochaetes OTUs from the Vhembe termite dataset (*n* = 10) as well as from the previously published Hadza 16S rRNA V4 gut microbiome dataset (*n* = 7) [48] and Shuar 16S rRNA V4 gut microbiome dataset (*n* = 8) [65] were aligned to the reference sequences, trimmed to the V4 hypervariable region, and used to construct a maximum likelihood (ML) tree (Fig. 6). The full length 16S genes of the reference sequences were similarly used to construct an ML tree to confirm the topology (Additional file 12: Figure S10). For both trees, the reference sequences are color coded based on their environmental occurrence or pathogenicity, which illustrates that nonpathogenic strains form clusters that are distinct from pathogenic strains, and that animal host-associated strains separate from environmental or termite host-associated strains, as has been demonstrated previously [23, 66]. Most of the Vhembe Spirochaetes OTUs cluster among *Treponema* strains that are environmentally sourced (*T. stenostreptum* and *T. caldarium*) or termite sourced (*T. primitia*, *T. isoptericolens*, and *T. azotonutricium*) while the Hadza Spirochaetes OTUs cluster among nonpathogenic porcine and ruminant gut symbionts. One notable exception stands out whereby a Vhembe *Treponema* OTU clusters with Shuar and Hadza *Treponema* OTUs, indicating that certain *Treponema* strains may be shared among diverse animal host groups given a shared environmental reservoir. In general, it appears that the majority of *Treponema* strains found within the termite gut microbiome are not associated with strains that inhabit or infect higher order animals, suggesting alternate and anachronistic modes of acquisition of these human and termite gut symbionts.

## Discussion

Recognition of the importance of insects in the human diet has had a slow but permeating effect on interpretations of dietary ecology in human evolution. As the Western ethnocentric bias on cultural conventions and taboos abates, and the need for sustainable food crops becomes more urgently felt, a greater awareness has emerged as to the cross-cultural biodiversity of human food resources. Entomophagy is a definitive human dietary trait, linked to deep primate evolutionary origins, but one that has been forgotten or rejected by the established culinary habits dominating the post-industrial cultural west. Insect foraging is primarily concentrated on five orders within Anthropoda, including Hymenoptera, Coleoptera, Lepidoptera, Orthoptera, and Isoptera, encompassing species of wasps, bees, ants, beetles, butterflies, moths, crickets, grasshoppers, locusts, and termites [67]. Since termites are a key resource both for modern and ancient hominids (humans and other great apes), we sought to open an investigation into edible termites, starting with characterization of the gut microbial community of two edible *Macrotermes* species.

The source of putatively non-pathogenic *Treponema* species observed in gut microbiome of tradition people, but absent industrialized people, has been of major interest to human microbiome research [46–50]. Phylogenetic analysis of *Treponema-*characterized termite-associated taxa shows separation from both pathogenic and non-pathogenic mammalian-associated taxa [23, 66] and their functions are given to xylan degradation [64], making the microbiome of edible termites an attractive target for illuminating the source of human-associated treponemes. Our study largely supports the phylogenetic characterization for *Treponema*, even for termites consumed by humans; however, uncertainties remain. Our analyses do find that some of our novel termite-associated *Treponema* fitting outside of the clade that harbors both the mammalian-associated and insect-associated taxa. This suggest that the phylogenetic picture of host-associated Treponema is far from fully resolved. Moreover, one of the Vhembe *Treponema* strains falls into a cluster with other human-derived Treponema strains (Hadza and Shuar). Thus, it is possible that there are multiple reservoirs of *Treponema*, and given functional redundancies of bacteria, termites may be one source for some mammals (like humans) that tap into these reservoirs. Similarly, termites that are favored to be consumed by humans may have a microbial complement that distinguishes themselves from termites that are less palatable to humans.

Until now there has been little attention as to how insect consumption affects consumer physiology from the standpoint of the gut microbiome. Recent work on white-faced capuchins shows that the capuchin gut microbiome composition is significantly impacted by invertebrate consumption, rather than by fruit consumption [68]. As an animal food resource, insect invertebrates are uniquely consumed whole and are themselves host to complex microbial communities. Therefore it stands to reason that insect gut microbial communities could in fact be an important source of the observed pattern of microbial alterations in the primate gut, and must be explored to understand their potential contributions. Early proto-primates were likely insectivorous mammals, much like today’s mouse lemurs, lorises, tarsiers, and galagos, but entomophagy is still sustained to some degree in larger-bodied monkeys and apes, including humans [5, 69, 70]. A speciose hominin lineage throughout the Plio-Pleistocene is owed in part to dietary niche diversity, in which shifting C3-C4 resource availability during climatic cooling required *Australopithecines* to adapt to challenging fallback foods that were likely high in cellulose and lignocellulose [71, 72]. The high-breadth diet model for members of *Homo* would similarly include the occasional need to process fibrous low-calorie plant foods [73]. The bacterial and protozoan communities of various wood feeding termite species are known to contain diverse genes for cellulose and xylan hydrolysis [19, 20, 64], and the microbial metagenomic specialization of the fungus-farming Macrotermitinae demonstrates presence of genes for oligosaccharide degradation [22]. Both metabolic strategies would have potential benefit for hominin consumers for digesting lignocellulose or secondary metabolism of oligosaccharides and CO_2_ into useful metabolites [19]. Modern human communities in the Limpopo region of South Africa preferentially harvest the major soldiers from two *Macrotermes* species, and similar selective behavior is observed among chimpanzee termite foragers [38]. The implications of this selectivity for gut health are provocative in light of anecdotal accounts that termite consumption alleviates symptoms of gastrointestinal discomfort among the Limpopo villagers. Accordingly, beneficial effects could be the result of a number of influences relating to chemical composition of termites as well as microbial assimilation and activity: digestion of fibrous foods; antidiarrheal treatment [5]; purging intestinal parasites; binding and purging toxins and anti-nutrients [42]; conferring prebiotic substrates; balancing pH, reducing sulfates; or supplementing essential minerals [5, 74].

### Diversity measurements reflect behavior

Contrary to our expectations, the alpha diversity of minor soldiers was significantly higher for the Observed Species metric, and slightly higher in the Phylogenetic Diversity metric than that of major soldiers of *M. falciger* and soldiers of *M. natalensis*. However, significant difference is seen only in the Observed Species metric, indicating that similar types of closely related microbial taxa populate at different frequencies within *M. falciger* and *M. natalensis* soldiers [75]. Since minor soldiers accompany and defend workers during forage and activities in the nest, then perhaps they have more exposure to environmental and food substrate that promotes more bacterial growth in the gut. Major soldiers are too large and cumbersome to chase threats or venture far from the nest [76], but they make good tunnel blockades against intruders (see Additional file 3: Figure S1 for size comparison), a behavioral feature similar to that seen for the ‘supersoldier’ subcaste of *Pheidole obtusopinosa* ants [77]. Nevertheless, *Macrotermes* lack gut compartmentalization, and sterile castes obtain their essential nutrition from fungus comb, which likely restrains any great deviation in abundance of phylogenetically diverse gut microbiota (Fig. 1) [26–28]. Comparison of taxonomic diversity between specimens (beta diversity) consistently shows that major and minor soldiers differentiate both in abundance (Bray-Curtis PCoA) and occurrence (unweighted UniFrac PCoA) of taxa (Fig. 1b), though batch effects cannot be discounted. *M. falciger* minor soldiers have the greatest spread on the plot area, and intersperse with *M. natalensis* soldiers, indicating similarities in their behavior, possibly from heightened affiliation with the worker castes.

### Resolving taxonomic differences

The indicator species analysis has been effectively co-opted for microbiome research, and was helpful in finding differentiating OTUs in our dataset. Certain taxa are very highly and very specifically associated to the termite soldier castes and species [45], providing incentive to delve further into a more appropriate method of characterization. Researchers previously overcame issues in taxonomic resolution by developing DictDb, a curated database of dictyopteran-specific gut microbiota [26] with good success. However our attempt with DictDb resulted in the majority of reads unclassified, potentially due to problems in implementation with different aligners. Use of abundance associations therefore allowed us to network and visualize broader taxonomic clusters that serve to characterize caste and species differences. In general, the differences among *Macrotermes* soldiers are mostly limited to abundance variation, with only a few taxa differentiating these soldiers by strict presence or absence. There is a visible tradeoff in enrichment between the *Alistipes* and *Ruminococcaceae* CAGs (left-hand side) for *M. natalensis* and *M. falciger* soldiers, and then a tradeoff between *Desulfovibrio/Alistipes* taxa and *Methanobrevibacter* taxa when comparing *M. falciger* major and minor soldiers. Curiously, high abundance of methanogenic *Methanobrevibacter* archaea in *M. falciger* major soldiers is replaced in *M. falciger* minors and *M. natalensis* soldiers by enrichment in Deltaproteobacteria families that are known sulfate reducing bacteria (SRB), including *Desulfovibrio*. Only under specific sulfate deplete conditions can methanogens completely outcompete SRB, where lactate fermentation tends to proceed to acetate by acetogens (such as Clostridia bacteria) and methane may serve as an electron sink for acetoclastic methanogens [56]. When sulfate is present, even at a ratio of 0.5 to lactate, the SRB dominate methanogens in abundance. However, nitrate can also serve as substrate for SRB [78], and the conidia supplied by the *Termitomyces* fungus are rich in nitrogen, which may explain the abundance of SRB in *M. falciger* minor soldiers and *M. natalensis* soldiers. Since the *M. falciger* major soldiers are fed by trophallaxis by older workers, who themselves feed exclusively on old fungal comb rather than the conidia, the major soldiers may lack sufficient sulfate or nitrate in their food substrate to prevent methanogen dominance over SRB. Since methane production is an undesirable consequence of raising animal products for human consumption, knowledge of the factors leading to methane production in edible termites may stimulate interest in developing sustainable systems of termite production that are easily implemented, environmentally responsible, and can empower marginalized economic actors [79].

### Fungus-farmers have atypical termite microbiomes

Our microbiome metaanalysis from several different termite species shows a broad division between wood- and soil-feeding termites on the one hand and the fungus-farming and food generalists of Blattodae on the other, matching previous findings [23, 27, 28]. The former, which comprise both higher and lower termites, are predictably sparse in co-abundant bacterial taxa diversity and are mainly dominated by few symbionts, primarily *Treponema*, with some specific contributions from *Tyzzarella*, *Synergistaceae*, *Ruminococcaceae*, and Bacteroidales (Fig. 5 and Additional file 7: Figure S5) [20]. Alongside eukaryotic flagellates in the lower termites, the bacteria found in wood- and soil-feeding termites are specialized to breakdown the large organic particles ingested by the termites, and require compartmentalization of the gut into chambers that maintain a strict alkaline condition or house morphological and biotic features such as cuticular spines and the flagellate protists [20]. The fungus-farming Macrotermitinae genera analyzed here (*Macrotermes* and *Odontotermes*) instead have a more generalized microbial profile that has been described as “heterogeneous” [80] and having a “relatively uniform composition” [28] and is comparable to the generalist-feeder cockroaches and even the scarab beetle (Additional file 11: Figure S9). Rather than use enteric microbial symbionts to decompose wood or plant litter, the Macrotermitinae feed organic matter to a domesticated *Termitomyces* fungus (each colony has its own variety) that grows in cultivated fungal gardens within the mound. The *Termitomyces* in turn provides the termites a more refined and nutritionally distilled food. Mature fungus comb produces nitrogen-rich conidia nodules that support growth for the young termites, while the older termites feed on old fungus comb itself, obtaining a mix of carbohydrates, alkanoic acids, phenols, sugars, and protein [81]. Of all termites, the *Macrotermes* have the most complex social organization in their caste-based division of labor. The complexity of *Macrotermes* and other fungus-farmer polyethism is reflected in their gut microbiome variation visualized across PCo2 of Fig. 4, replicating similar findings reported in [24]: the Fig. 3 dendrogram shows a wide spread of 16S profiles for within *Macrotermes gilvus* specimens compared to the wood-feeding *Reticulitermes* spp. Not surprisingly, polyethism-driven feeding differences of Macrotermitinae confer a high degree of within-species gut microbiome variation in comparison to other non-fungus-farming termites (Fig. 4) [45].

### Implications for microbe-host relationships

Critically, the variation seen in the gut microbiome within a single *Macrotermes* colony (this study and in [24, 45]) are examples of where the notion of co-speciation and stabilization of a colony-specific microbiome [23] are not upheld [27]. Host phylogeny as an explanation of termite microbial patterns breaks down when dietary non-specialists are considered, indicating that host genetics and vertical inheritance are only relevant up to a certain point, after which ecological factors such as dietary niche best explain microbiome assimilation [82]. Intra- and inter-community variation within a termite species, and different degrees of variation seen between species, indicates that the termite colony environment, behavior, and resource access overwhelmingly influence gut microbiome features. The extent to which humans and great apes are selective about what termite mounds to target and even the particular caste type to eat [38] illustrates how ecological and behavioral nuances impart wide ranging biological variation, including to the nutritional and microbial contents. Recent work showing core taxonomic membership in global soil microbiomes, similar to that seen among world-wide traditional human groups [63], further dispels a general co-evolutionary hypothesis of gut microbiome acquisition [83]. Instead, microbial membership appears conserved based on habitat, not lineage, and permeates the environmental backdrop of all ecosystems, especially through soil. Soils cultivate microbial systems based on biotic and abiotic factors such as pH, aridity, productivity, and plant life [83], just as animals cultivate microbiome communities through morphology, physiology, diet, sociality, and environmental interaction, which are necessarily convergent phenotypic and niche properties for unrelated organisms around the world.

## Conclusion

We found significant alterations in the taxonomic abundance of dominant microbiota between soldier castes within *Macrotermes* species, as well as between soldiers from two different *Macrotermes* species. We also show that the microbial co-abundant groups of fungus-farming Macrotermitinae display a pattern of enrichment that mainly involves the *Alistipes* and *Ruminococcaceae* CAGs, whereas the wood- and soil-feeding termites are characterized by a high abundance of Termite *Treponema* cluster. Macrotermitinae co-abundant taxa are more disperse and align closest to the dietary generalist sister clade of non-eusocial cockroach taxa, corroborating previous findings based on functional and taxonomic comparisons [22, 23]. Phylogenetic analysis of *Treponema* OTUs from edible termites demonstrates that termite-associated *Treponema* taxa are mostly separate from both pathogenic and non-pathogenic mammalian-associated taxa with one exception, suggesting that additional reservoirs of *Treponema* diversity could be supplied to humans through a shared environmental vector with termites, like soil, or via consumption of termites directly. Though, termite *Treponema* still mainly cluster among other environmentally sourced treponemes [26].

Human consumption of insects represents one such culturally and regionally variant dietary niche that is nutritionally significant for (but not exclusive to) many impoverished, disenfranchised, or rural subsistence-based communities. Insects provide high quality protein and fat that often supplements an otherwise lower quality plant-based diet. The management of insect harvesting can service a number of topical issues, including economy and food security, but much work remains towards ensuring that environmental responsibility and equal economic opportunities are not sacrificed [3]. As revealed in our study, that management would benefit by deeper understanding of the nuances of termite ecology and human selection, perhaps helping to incentivize broader cultural acceptance of termites as food. Overall, there is substantial diversity in the overall community profile, which appears more predicted by behavior/environmental factors than host phylogeny, an understanding this is likely to be a valuable consideration for future efforts in management and exploration of health impacts.

## Methods

### Collection

Termites were collected whole in collaboration with local peoples from a small village in the Limpopo province in the northeastern region of South Africa. Two recognized edible termites were targeted for this study, taxonomically verified as *Macrotermes falciger* (larger species) and *Macrotermes natalensis* (smaller species). The larger of these, *M. falciger*, are commonly found at local markets in the region, while the smaller *M. natalensis* are not found in the market. For *M. falciger*, a major (large) and minor (small) soldier is commonly identified, while for *M. natalensis* caste differences among soldiers are not readily apparent. Only the major specimens of *M. falciger* are selected for sale in local markets. All soldiers though are edible, and *M. natalensis* soldiers are not filtered before consumption. For the purposes of this research, all soldier types (hereon referred to as “castes”) were collected from *M. falciger* and *M. natalensis* wild mounds (*n* = 8 mounds) found near to a small village in Limpopo. Termites collected in the field were immediately submerged in 80% ethanol until shipment. For shipment, specimens were sealed whole into glass jars along with cotton balls dipped in 80% ethanol and shipped express to the Laboratories of Molecular Anthropology and Microbiome Research in Norman, Oklahoma, USA. Upon arrival to the laboratory, all samples were frozen at − 20 °C for long-term storage until further use.

### Dissection and sampling

To target the microbiota of the alimentary tract, whole termites were dissected following steps 1–2 of an extraction protocol described previously [53]. The goal of this procedure was to isolate the entire gut from the rest of the termite body for use in downstream extraction methods, with an interest in sampling the microbiota from the whole gut, not just gut segments. Dissections were conducted using a dissection microscope and stainless steel, extra-fine, curved microdissection forceps (Carolina Biological Supply Company). Termite specimens were held supine at the head-thorax junction with utility forceps, and the distal end of the abdomen was pinched with micro forceps and pulled in a continuous motion to remove the entire gut tract (Additional file 3: Figure S1). Termites were kept on ice immediately prior to dissection and dissected gut tracts were placed into a microcentrifuge tube containing 50 μl TE buffer (1 mM Tris-HCl, 0.1 mM EDTA, pH 8.0) per each individual gut and macerated with forceps. Individual gut weights were obtained on a tarred microbalance, and then specimens were stored at − 20 °C until further use. After each dissection, forceps were washed in a 20% v/v bleach-water solution (6% sodium hypochlorite bleach solution, deionized-water), rinsed with water to remove residual bleach, and then dried with a Kimwipe dampened with a 70% ethanol solution. The stage was also rinsed and wiped with 70% ethanol solution after each dissection to maintain a sterile working environment and reduce chance of cross-contamination between specimens (see Additional file 3: Figure S1 for images of the dissection workstation).

To validate our ability to capture representative ecologies from single termite guts, we set up one experiment to compare single gut extractions with pooled, fractioned, and fractioned/pooled. Thirty-two *M. falciger* from the mound, Vhembe 4, were dissected (16 major, 16 minor), and the full GI-tract removed, weighed, and immediately stabilized. Of the 16 × 2 dissected guts (one set of 16 for each caste) *n* = 5 were extracted singly (S), *n* = 6 were pooled in two groups of three (P), and n = 5 were macerated and fractioned 50% by volume (F). The second half of the five fractioned guts were pooled together in a single “pooled fraction” (cF), resulting in a total of 13 × 2 = 26 extracted samples for this batch run.

### Extraction and quantification

DNA extraction of termite guts was accomplished using the MoBio PowerSoil® kit using a slightly modified protocol. Each dissected gut in TE buffer received 20 μl of Proteinase K (Qiagen) and was the incubated on a shaker for 8–12 h at 55 °C and 150 rpm alongside negative controls containing 50 μl TE buffer and 20 μl Proteinase K in a microcentrifuge tube. After incubation, samples and negative controls were transferred quantitatively to bead tubes containing 0.7 mm garnet and PowerSoil® bead solution (which contains aqueous guanidine thiocyanate) and 60 μl of solution C1. Bead beating was carried out on a Vortex Genie2 at maximum speed for 10 min, after which tubes were spun down at 8000 x g for 1 min. The MoBio PowerSoil® extraction kit was subsequently used for purification and elution. Samples and negatives were eluted in 100 μl of solution C6, and total DNA concentration measured on a Qubit Fluorometer using the high sensitivity assay (see Additional file 1: Table S1). All negatives were below detection level. Elutions were stored at − 20 °C until further use. To quantify bacterial DNA and determine the appropriate minimum number of amplification cycles, quantitative real-time PCR (qPCR) was conducted using the FastStart Essential DNA Green Master (Roche) and 16S rRNA gene primers 515F/806R for the V4 hypervariable region. Reactions were conducted in 25.0 μl, with 12.5 μl FastStart Essential master mix, 0.75 μl each of 10 μM forward and reverse primers, 1.0 μl template DNA, and 10 μl nuclease-free PCR-grade water. Reactions without template DNA served as PCR negative controls alongside the extraction negatives, and *Echerichia coli* DNA was used as a positive control and quantitative reference. Cycling was done on a Roche LightCycler® 96 with the following program: 600 s at 95 °C; then 45 cycles of 10 s at 95 °C, 20 s at 52 °C, and 30 s at 72 °C. Samples that successfully amplified had Cq-values within a range of 15–23 while negative controls were > 35, indicating negligible influence from contamination below 30 cycles. Gel electrophoresis confirmed that amplified DNA fell within the size range expected for the targeted V4 region of bacterial DNA (~ 400 bp) and was not likely of host origin.

### Amplification and sequencing

Amplification of the V4 hypervariable region of the 16S rRNA gene was conducted using the bacterial-archaeal 515F/806R primers with Illumina adapters [84], which contain unique 12 bp 2168 GoLay barcodes on the reverse primer: forward construct - AATGATACGGCGACCACCGAGATCTACAC TATGGTAATT GT GTGCCAGCMGCCGCGGTAA; reverse construct - CAAGCAGAAGACGGCATACGAGAT [12 bp unique barcode] AGTCAGTCAG CC GGACTACHVGGGTWTCTAAT. Platinum *Taq* (Invitrogen) was used to amplify the majority of the samples (*n* = 57) as well as the positive and negative controls. The amplification was carried out in a 15 μl volume containing 2.4 μl dNTPs (2 mM), 1.5 μl BSA (2.5 mg/ml), 0.9 MgCl_2_ (50 mM), 1.5 μl 10x PCR buffer, 0.36 μl forward primer (10 μM), 1.44 μl reverse primer (2.5 μM), 0.1 μl Platinum *Taq*, 1.5 μl template DNA, and 5.3 μl nuclease-free PCR-grade water. Cycling conditions consisted of initial denaturation at 98 °C for 120 s and 25 cycles of 98 °C for 20 s, 52 °C for 30 s, and 72 °C for 30 s, followed by a final elongation at 72 °C for 300 s. A batch of samples from a single mound (*n* = 10) were amplified using KAPA HiFi DNA polymerase in a 25.0 μl reaction volume containing 1.0 μl MgCl_2_ (25 mM), 1.0 μl bovine serum albumin (BSA; 2.5 mg/ml), 0.75 μl forward primer (10 μM), 3.0 μl reverse primer (2.5 μM), 12.5 μl KAPA HiFi HotStart ReadyMix, 4.0 μl template DNA, and 2.75 μl nuclease-free PCR-grade water. Cycling conditions consisted of initial denaturation at 98 °C for 120 s and 25 cycles of 98 °C for 20 s, 48 °C for 30 s, and 72 °C for 30 s, followed by a final elongation at 72 °C for 300 s. For all batch runs, the same positive control sample was used, which derived from a single human fecal sample extraction.

Amplifications were conducted in triplicate and gel electrophoresis was used to confirm presence of the expected amplicon. All replicates for a sample were then pooled, run on a 2% agarose gel, visualized using the Vision Works Software, and quantified using 1D-analysis. A 150 ng aliquot from each amplified sample was pooled along with 1 μl of the positive control and 5 μl of the negative controls. A 250 μl aliquot of the pool was purified using a MinElute PCR purification (Qiagen) and the eluate was size selected with Pippin Prep and quantified with the Fragment Analyzer (Advanced Analytical). The samples and controls were sequenced over three paired-end 2 × 250 bp runs on an Illumina platform (NextSeq and MiSeq).

### Bioinformatics

#### In-house generated data

Sequence data were demultiplexed using Illumina’s bcl2fastq and read pairs merged using PEAR [85] with a minimum overlap (−v) 50, minimum assembled length (−t) 150, maximum assembled length (−m) 270, minimum quality score (−q) 30, and maximum uncalled bases (−u) 0. Resulting FASTQ files were quality filtered using USEARCH fastq_filter [86] with maximum expected error rate set to 0.5. Resulting FASTA files for each run were combined and processed using a suite of commands from the USEARCH software and QIIME scripts [87, 88] in a workflow as follows: USEARCH dereplication and sorting by size; denovo operational taxonomic unit (OTU) clustering using USEARCH UPARSE algorithm [89] with minimum size = 5 to remove spurious reads - this call also removed chimeras; USEARCH OTU table creation with the global search of OTUs on the original combined FASTA file, with the identity set to 0.97; OTU sequences aligned using MUSCLE [90]; phylogenetic tree built from aligned OTUs using the FastTree tree alignment tool implemented in QIIME [91]; alpha-diversity metrics - observed species and Faith’s phylogenetic diversity [92] - calculated on multiple rarefactions of the OTU table up to a read depth of 8000 using QIIME; taxonomy assigned for denovo clustered OTUs using the default uclust assigner implemented in QIIME against the SILVA representative taxonomy, release 132 [55]; taxonomy annotated OTU table rarefied to a single depth of 8000 reads to create the final working OTU table for downstream analysis; summarize taxonomies and UniFrac [93] beta-diversity calculations created from the rarefied OTU table using QIIME scripts. Of note, we attempted to use the DictDb [26] database for taxonomic assignment, however the database was not compatible with our choice of alignment program.

#### Reference sequences

Reference termite gut microbiome sequence data were downloaded from NCBI SRA for three different studies [23, 45, 60] and NCBI BioProject accession PRJNA315033 (Additional file 1: Table S1). All but one of these external datasets were generated with 454 pyrosequencing, and so required slightly different bioinformatic procedures. First, FASTQ files were visualized using FastQC [94] to assess quality score distributions and linker/primer/adapter/barcode content. Cutadapt [95] was used to remove non-sequence regions, trim low-quality 3′ bases, and remove reads shorter than 200. In the case of one study dataset [23] that used bidirectional 454 sequencing, the sequences were parsed for sense and anti-sense forward and reverse reads using the forward and reverse primers, binned separately, and then Cutadapt used to remove primers and trim low quality ends on binned reads. After these trimmed FASTQs were converted to FASTAs using USEARCH fastq_filter, the anti-sense reads were reverse complimented and concatenated to the sense reads to create one merged FASTA file. For all other datasets, USEARCH fastq_stats informed the average expected error of reads for each dataset, and then FASTQs were filtered with USEARCH fastq_filter to create FASTA files. Denovo OTU picking was conducted for all study FASTA files separately as described above using USEARCH UPARSE, but with a minimum unique sequence size = 2. Taxonomy was again assigned with SILVA as described above, and the resulting OTU tables were rarefied individually to the lowest sequencing depth required to retain at least 80% of samples, but no lower than 1000 reads, and taxonomy summaries created using QIIME. Samples that were excluded due to low final read count or low read assignment are noted in Additional file 1: Table S1. Finally, the genus level (L6) summarized taxa tables from each dataset were merged using the merge_OTU_tables.Py script in QIIME, and the resulting merged table file used for all downstream analyses

### Analysis and statistics

All statistical analyses were conducted in R version 3.4.1 [96]. Several packages were used alongside base {stats} and {graphics}. Procrustes rotation, beta-dispersion, rarefaction, ordination, clustering, and permutational multivariate analysis (PERMANOVA) of variance were conducted with {vegan} [97]. Heatplots were generated using {made4} [98]. Data frames were reformatted using {reshape2} [99]. Indicator species analysis was conducted using {labdsv} [100]. Kendall correlation tau distance was computed using {bioDist} [101]. The Benjamini-Hochberg method was used for multiple testing corrections, with false discovery rate (FDR) < 0.05 considered as statistically significant to reduce the rate of type-I errors.

#### Co-abundance group networks

Co-abundance network plots were generated using Cytoscape 3.5.1 [102] using the taxonomy summary L6 table generated by QIIME, filtered for taxa abundant at 0.1% in at least 30% of samples. Co-abundance groups (CAGs) were created by first evaluating the associations among genera using the Kendall correlation test using the base “cor” function in R with FDR corrected *p*-values, creating a correlation matrix of the taxa abundances. Next, these correlations were visualized using hierarchical Ward clustering with a Spearman correlation distance metric (e.g. 1-cor(x)), and groups annotated using “cutree” in {vegan}. The appropriate number of co-abundance groups that best explains the clustering of the taxa were selected based on significance testing among each group on the original Kendall correlation matrix, which was converted into a distance matrix using “tau.dist” in {bioDist}, using “adonis” in {vegan}. Significant associations were controlled for multiple testing with FDR. Finally, once CAGs were defined, then two tables were created for import into Cytoscape: 1) a network (edges) dataframe that lists all pairwise combinations of taxa (source and target) and their relationship value (correlation coefficient); and 2) a metadata dataframe that defines the node characteristics (list of taxa and their CAG group and relative abundance value). These files were imported into Cytoscape and the Compound Spring Embedder (CoSE) layout (a modification of the force-directed layout) selected for representation of the network.

#### Indicator species

Indicator species (IS), defined by a value from the product of the relative frequency and relative average abundance among a pre-defined group of samples, were calculated using the {labdsv} package on the rarefied OTU table. Values greater than 0.6 were considered as meaningful IS (i.e. OTUs), with the range from 0 to 1. OTUs meeting the indicator value cut-off were correlated by Kendall rank correlation and visualized in a heatplot using the Spearman distance of the tau correlation coefficients. Log2 fold change of the group mean relative abundance of OTUs was used to illustrate the differences in IS abundance between pair groups (either between major and minor caste or between *M. falciger* and *M. natalensis* species). A Wilcoxon test determined whether abundance differences between groups were significant (FDR corrected p-value < 0.05 considered statistically significant). In order to account for potential run batch bias, those OTUs that received an IS score > 0.6 among run comparisons were first removed from consideration for caste-based comparisons using the combined dataset. Caste-based comparisons for IS analysis were done for *M. falciger* samples combined from all three runs. Termite species-based comparisons for IS analysis were conducted using only the Run3 dataset, rather than combined dataset.

#### Treponema phylogenetic analysis

Complete 16S rRNA gene sequences of *Treponema* and *Brachyspira* species (both genus-level members of the Spirochaetes phylum) were acquired from the NCBI RefSeq database. These reference sequences were aligned using MAFFT v7.271 [103] with default parameters and the “--adjustdirectionaccurately” option. Positions with less than 95% coverage were eliminated, resulting in a total of 1326 positions in the final analysis. A maximum likelihood (ML) tree was built in MEGA [104] using the Kimura 2-parameter model with gamma distribution and invariant sites to allow for evolutionary rate heterogeneity among sites. This model was chosen because it was the best-fit model according to MEGA’s Model Test. Bootstrap support was estimated from 500 replicates. To model the phylogenetic relationship among the Spirochaetes OTUs from our V4 16S rRNA gene data, the OTU reads assigned to the Spirochaetes phylum were acquired from the OTU FASTA file and merged into a separate FASTA file. Additionally, the Spirochaetes-assigned OTUs from two other gut microbiome datasets from human hunter-gatherer populations, Shuar of Ecuador and Hadza of Tanzania [48, 65], were also included. These OTU representative sequences were aligned to the reference *Treponema* and *Brachyspira* sequences using MAFFT. The alignment was trimmed to the V4 region and gaps and missing data were eliminated, resulting in a total of 253 nucleotide positions in the final analysis. An ML tree was built using the Kimura 2-parameter model with gamma distribution and invariant sites to allow for evolutionary rate heterogeneity among sites. Bootstrap support was estimated from 500 replicates; values above 50% are annotated in the final tree.

## Additional files

Additional file 1: **Table S1.** Sample metadata. All sample metadata information of this study data and external data used can be found here. (XLSX 30 kb)

Additional file 2: **Table S2.** Metadata correlation for Run2 sample taxonomy. Correlations for Run2 samples between metadata variables (gut mass, DNA concentration in extraction, qPCR cq value, and diversity metrics) and taxonomic abundance summarized at the genus (L6) level. Significant values after FDR correction highlighted in yellow (XLSX 20 kb)

Additional file 3: **Figure S1.** Termite dissection and inventory images. The dissection space and sample documentation shows the set up for sterile sample handling as well as comparisons of termites and examples of a dissected gut. (PDF 2672 kb)

Additional file 4: **Figure S2.** Ecology verification with Run2 samples. (A) Procrustes rotation of 14 k and 1 k rarefied OTU table. (B) Procrustes rotation of single versus pooled and single versus fractioned samples. (C) Bray-Curtis ordination and heatplot of all Run2 samples shows no apparent clustering by extraction type. (PDF 518 kb)

Additional file 5: **Figure S3.** Metadata correlation of Run2 dissection and extraction data and taxonomic abundance. Dissection and extraction variables do not indicate biases in alpha-diversity metrics based on gut mass, extraction concentration, and qPCR Cq values. Taxonomic bias is also not apparent. (PDF 421 kb)

Additional file 6: **Figure S4.** Batch analysis using control samples. (A) Positive gut ecology controls cluster together. (B) Ward clustering confirms ordination. (C) Additional diversity characterization from rarefaction curves and diversity metrics shown by termite mound. (PDF 388 kb)

Additional file 7: **Figure S5.** Phylum and genus level taxonomy summaries. (A) Taxonomy summaries at Phylum and Genus level for in-house Vhembe termite data, with color key indicating the run, termite type, and mound, respectively (color key as in Fig. 1). (B) Taxonomy summaries for reference sequence data and in-house data. (PDF 1357 kb)

Additional file 8: **Figure S6.** Co-abundance group clustering and network. Correlation heatplot of filtered taxa for in-house samples made with Kendall correlation of abundance and clustered using Ward’s method with Spearman distance. Co-abundance groups were imported into Cytoscape software to visualize the network. Nodes are sized by mean taxonomic abundance and edge widths indicate correlation coefficient. (PDF 494 kb)

Additional file 9: **Figure S7.** Reference data meta-analysis NMDS. NMDS plot showing stress value and Shepard plot indicating fit. (PDF 849 kb)

Additional file 10: **Figure S8.** Co-abundance groups for reference meta-data analysis. Three co-abundance groups were found using the same procedure, and visualized in the network. (PDF 316 kb)

Additional file 11: **Figure S9.** CAGs for other invertebrate data. Scarab beetle larvae CAG best resembles termite fungus farmers and cockroach CAG profiles. (PDF 213 kb)

Additional file 12: **Figure S10.** Spirochaetes maximum likelihood tree of full 16S rRNA gene sequences of reference taxa from NCBI. Full length 16S rRNA gene sequences of reference Spirochaetes were first aligned and analyzed to view the sequence relationships using more sites. The percentage of trees in which the associated taxa clustered together is shown next to the branches. Branch labels are color-coded based on strain association to source and pathogenicity: red - pathogenic *Treponema*; purple - non-pathogenic *Treponema*; green - environmental *Treponema*; blue - *Treponema* species associated with termite gut. (PDF 197 kb)

## Data Availability

Sequence data generated and analyzed during the current study are available in the NCBI SRA, BioProject ID: PRJNA436004, Submission ID: SUB3727452; http://www.ncbi.nlm.nih.gov/bioproject/436004 Supplementary metadata and pairwise correlations can be found in Additional file 1: Table S1 and Additional file 2: Table S2 respectively. Supporting figures can be found in Additional file 3: Figure S1, Additional file 4: Figure S2, Additional file 5: Figure S3, Additional file 6: Figure S4, Additional file 7: Figure S5, Additional file 8: Figure S6, Additional file 9: Figure S7, Additional file 10: Figure S8, Additional file 11: Figure S9, Additional file 12: Figure S10.

## References

[1] Milton K, Harris M, Ross E (1987). Primate diets and gut morphology: implications for hominid evolution. Food and evolution.

[2] Bown TM, Gingerich PD (1973). The Paleocene primate Plesiolestes and the origin of Microsyopidae. Folia Primatol.

[3] Payne CLR, Van Itterbeeck J (2017). Ecosystem services from edible insects in agricultural systems: a review. Insects..

[4] Redford KH (1984). The nutritional value of invertebrates with emphasis on ants and termites as food for mammals. J Zool.

[5] Deblauwe I, Janssens GPJ (2008). New insights in insect prey choice by chimpanzees and gorillas in Southeast Cameroon: the role of nutritional value. Am J Phys Anthropol.

[6] D’Errico F, Backwell LR, Berger LR (2001). Bone tool use in termite foraging by early hominids and its impact on our understanding of early hominid behaviour. S Afr J Sci.

[7] Sponheimer M, Lee-Thorp J, de Ruiter D, Codron D, Codron J, Baugh AT (2005). Hominins, sedges, and termites: new carbon isotope data from the Sterkfontein valley and Kruger National Park. J Hum Evol.

[8] Boutton TW, Arshad MA, Tieszen LL (1983). Stable isotope analysis of termite food habits in east African grasslands. Oecologia..

[9] Pobiner BL, Rogers MJ, Monahan CM, Harris JWK (2008). New evidence for hominin carcass processing strategies at 1.5 Ma, Koobi fora, Kenya. J Hum Evol.

[10] Speth JD, Spielmann KA (1983). Energy source, protein metabolism, and Hunter-gatherer subsistence strategies. J Anthropol Archaeol.

[11] Marten A, Kaib M, Brandle R (2009). Cuticular hydrocarbon phenotypes do not indicate cryptic species in fungus-growing termites (Isoptera: Macrotermitinae). J Chem Ecol.

[12] Howard RW, Blomquist GJ (2005). Ecological, behavioral, and biochemical aspects of insect hydrocarbons. Annu Rev Entomol.

[13] Stull VJ, Finer E, Bergmans RS, Febvre HP, Longhurst C, Manter DK (2018). Impact of edible cricket consumption on gut microbiota in healthy adults, a double-blind, randomized crossover trial. Sci Rep.

[14] Paoletti MG, Norberto L, Damini R, Musumeci S (2007). Human gastric juice contains Chitinase that can degrade chitin. Ann Nutr Metab.

[15] Janiak MC, Chaney ME, Tosi AJ (2017). Evolution of acidic mammalian Chitinase genes (CHIA) is related to body mass and Insectivory in Primates. Mol Biol Evol.

[16] Hamad I, Delaporte E, Raoult D, Bittar F (2014). Detection of termites and other insects consumed by African great apes using molecular fecal analysis. Sci Rep.

[17] Bourguignon T, Lo N, Cameron SL, Sobotnik J, Hayashi Y, Shigenobu S (2014). The evolutionary history of termites as inferred from 66 mitochondrial genomes. Mol Biol Evol.

[18] Radek R (1999). Flagellates, Bacteria, and Fungi associated with termites: diversity and function in nutrition - a review. Ecotropica..

[19] Breznak JA, Brune A (1994). Role of microorganisms in the digestion of lignocellulose by termites. Annu Rev Entomol.

[20] Brune A, Dietrich C (2015). The gut microbiota of termites: digesting the diversity in the light of ecology and evolution. Annu Rev Microbiol.

[21] Wood TG, Thomas RJ (1989). The mutualistic association between Macrotermitinae and Termitomyces. Insect-fungus interactions.

[22] Poulsen M, Hu H, Li C, Chen Z, Xu L, Otani S (2014). Complementary symbiont contributions to plant decomposition in a fungus-farming termite. Proc Natl Acad Sci.

[23] Dietrich C, Köhler T, Brune A (2014). The cockroach origin of the termite gut microbiota: patterns in bacterial community structure reflect major evolutionary events. Appl Environ Microbiol.

[24] Hongoh Y, Ekpornprasit L, Inoue T, Moriya S, Trakulnaleamsai S, Ohkuma M (2006). Intracolony variation of bacterial gut microbiota among castes and ages in the fungus-growing termite Macrotermes gilvus. Mol Ecol.

[25] Makonde HM, Mwirichia R, Oslemo Z, Boga HI, Klenk H-P (2015). 454 pyrosequencing-based assessment of bacterial diversity and community structure in termite guts, mounds and surrounding soils. Springerplus..

[26] Mikaelyan A, Köhler T, Lampert N, Rohland J, Boga H, Meuser K (2015). Classifying the bacterial gut microbiota of termites and cockroaches: a curated phylogenetic reference database (DictDb). Syst Appl Microbiol.

[27] Mikaelyan A, Dietrich C, Köhler T, Poulsen M, Sillam-Dusses D, Brune A (2015). Diet is the primary determinant of bacterial community structure in the guts of higher termites. Mol Ecol.

[28] Otani S, Mikaelyan A, Nobre T, Hansen LH, Koné NA, Sørensen SJ (2014). Identifying the core microbial community in the gut of fungus-growing termites. Mol Ecol.

[29] Liu N, Zhang L, Zhou H, Zhang M, Yan X, Wang Q (2013). Metagenomic insights into metabolic capacities of the gut microbiota in a fungus-cultivating termite (Odontotermes yunnanensis). PLoS One.

[30] Aanen DK, Eggleton P, Rouland-Lefèvre C, Guldberg-Frøslev T, Rosendahl S, Boomsma JJ (2002). The evolution of fungus-growing termites and their mutualistic fungal symbionts. Proc Natl Acad Sci.

[31] Leuthold RH, Badertscher S, Imboden H (1989). The inoculation of newly formed fungus comb with Termitomyces in Macrotermes colonies (Isoptera, Macrotermitinae). Insect Soc.

[32] McGrew WC, Stanford CB, Bunn HT (2001). The other faunivory: primate insectivory and early human diet. Meat-eating and human evolution.

[33] Jongema Y (2017). Worldwide list of edible insects.

[34] Goodall J. My life among wild chimpanzees: National Geographic Society; 1963.

[35] Bukkens SGF (1997). The nutritional value of edible insects. Ecol Food Nutr.

[36] O’Malley RC, Power ML (2012). Nutritioinal composition of actual and potential insect prey for the Kasekela chimpanzees of Gombe national park, Tanzania. Am J Phys Anthropol.

[37] Rumpold BA, Schlüter OK (2013). Nutritional composition and safety aspects of edible insects. Mol Nutr Food Res.

[38] Lesnik JJ (2014). Termites in the hominin diet: a meta-analysis of termite genera, species and castes as a dietary supplement for south African robust australopithecines. J Hum Evol.

[39] Ruelle Jean-Emile (1970). A revision of the termites of the genus Macrotermes from the Ethiopian region (Isoptera : Termitidae). Bulletin of the British Museum (Natural History)..

[40] Darlington JPEC (2005). Distinctive fossilised termite nests at Laetoli, Tanzania. Insectes Soc.

[41] Brandle R, Hyodo F, von Korff-Schmising M, Maekawa K, Miura T, Takematsu Y (2007). Divergence times in the termite genus Macrotermes (Isoptera: Termitidae). Mol Phylogenet Evol.

[42] Krishnamani R, Mahaney WC (2000). Geophagy among primates: adaptive significance and ecological consequences. Anim Behav.

[43] Hunter JM (1993). Macroterme geophagy and pregnancy clays in southern Africa. J Cult Geogr.

[44] Netshifhefhe SR, Kunjeku EC, Duncan FD (2018). Human uses and indigenous knowledge of edible termites in Vhembe District, Limpopo Province, South Africa. S Afr J Sci.

[45] Li H, Dietrich C, Zhu N, Mikaelyan A, Ma B, Pi R (2016). Age polyethism drives community structure of the bacterial gut microbiota in the fungus-cultivating termite Odontotermes formosanus. Environ Microbiol.

[46] De Filippo C, Cavalieri D, Di Paola M, Ramazzotti M, Poullet JB, Massart S (2010). Impact of diet in shaping gut microbiota revealed by a comparative study in children from Europe and rural Africa. Proc Natl Acad Sci.

[47] Tito RY, Knights D, Metcalf J, Obregon-Tito AJ, Cleeland L, Najar F (2012). Insights from characterizing extinct human gut microbiomes. PLoS One.

[48] Schnorr SL, Candela M, Rampelli S, Centanni M, Consolandi C, Basaglia G (2014). Gut microbiome of the Hadza hunter-gatherers. Nat Commun.

[49] Obregon-Tito AJ, Tito RY, Metcalf J, Sankaranarayanan K, Clemente JC, Ursell LK (2015). Subsistence strategies in traditional societies distinguish gut microbiomes. Nat Commun.

[50] Ou Junhai, Carbonero Franck, Zoetendal Erwin G, DeLany James P, Wang Mei, Newton Keith, Gaskins H Rex, O’Keefe Stephen JD (2013). Diet, microbiota, and microbial metabolites in colon cancer risk in rural Africans and African Americans. The American Journal of Clinical Nutrition.

[51] Clemente Jose C., Pehrsson Erica C., Blaser Martin J., Sandhu Kuldip, Gao Zhan, Wang Bin, Magris Magda, Hidalgo Glida, Contreras Monica, Noya-Alarcón Óscar, Lander Orlana, McDonald Jeremy, Cox Mike, Walter Jens, Oh Phaik Lyn, Ruiz Jean F., Rodriguez Selena, Shen Nan, Song Se Jin, Metcalf Jessica, Knight Rob, Dantas Gautam, Dominguez-Bello M. Gloria (2015). The microbiome of uncontacted Amerindians. Science Advances.

[52] Gomez A, Petrzelkova KJ, Burns MB, White BA, Leigh SR, Blekhman R (2016). Gut microbiome of coexisting BaAka pygmies and bantu reflects gradients of traditional subsistence patterns. Cell Rep.

[53] Matson E, Ottesen E, Leadbetter J (2007). Extracting DNA from the gut microbes of the termite (Zootermopsis Angusticollis) and visualizing gut microbes. J Vis Exp.

[54] Paul K, Nonoh JO, Mikulski L, Brune A (2012). “Methanoplasmatales,” Thermoplasmtales-Related Archaea in Termite Guts in Other Environments, Are the Seventh Order of Methanogens. Appl Environ Microbiol.

[55] Quast C, Pruesse E, Yilmaz P, Gerken J, Schweer T, Yarza P (2013). The SILVA ribosomal RNA gene database project: improved data processing and web-based tools. Nucleic Acids Res.

[56] Dar SA, Kleerebezem R, Stams AJM, Kuenen GJ, Muyzer G (2008). Competition and coexistence of sulfate-reducing bacteria, acetogens and methanogens in a lab-scale anaerobic bioreactor as affected by changing substrate to sulfate ratio. Appl Microbiol Biotechnol.

[57] Stevens EC, Hume ID (1998). Contributions of microbes in vertebrate gastrointestinal tract to production and conservation of nutrients. Physiol Rev.

[58] Liu Y, Whitman WB (2008). Metabolic, phylogenetic, and ecological diversity of the methanogenic archaea. Ann N Y Acad Sci.

[59] Leadbetter JR, Breznak JA (1996). Physiological ecology of Methanobrevibacter cuticularis sp. nov. and Methanobrevibacter curvatus sp. nov., isolated from the hindgut of the termite Reticulitermes flavipes. Appl Environ Microbiol.

[60] Köhler T, Dietrich C, Scheffrahn RH, Brune A (2012). High-resolution analysis of gut environment and bacterial microbiota reveals functional compartmentation of the gut in Wood-feeding higher termites (Nasutitermes spp.). Appl Environ Microbiol.

[61] Yatsunenko T, Rey FE, Manary MJ, Trehan I, Dominguez-Bello MG, Contreras M (2012). Human gut microbiome viewed across age and geography. Nature..

[62] Martinez I, Stegen JC, Maldonado-Gomez M, Eren AM, Siba PM, Greenhill AR (2015). The gut microbiota of rural Papua new Guineans: composition, diversity patterns, and ecological processes. Cell Rep.

[63] Schnorr SL, Sankaranarayanan K, Lewis CM, Warinner C (2016). Insights into human evolution from ancient and contemporary microbiome studies. Curr Opin Genet Dev.

[64] Warnecke F, Luginbühl P, Ivanova N, Ghassemian M, Richardson TH, Stege JT (2007). Metagenomic and functional analysis of hindgut microbiota of a wood-feeding higher termite. Nature..

[65] Stagaman K, Cepon-Robins TJ, Liebert MA, Gildner TE, Urlacher SS, Madimenos FC (2018). Market Integration Predicts Human Gut Microbiome Attributes across a Gradient of Economic Development. mSystems.

[66] Ohkuma M, Iida T, Kudo T (1999). Phylogenetic relationships of symbiotic spirochetes in the gut of diverse termites. FEMS Microbiol Lett.

[67] McGrew WC (2014). The “other faunivory” revisited: Insectivory in human and non-human primates and the evolution of human diet. J Hum Evol.

[68] Mallott EK, Amato KR, Garber PA, Malhi RS (2017). Influence of fruit and invertebrate consumption on the gut microbiota of wild white-faced capuchins (Cebus capucinus). Am J Phys Anthropol.

[69] Pickett SB, Bergey CM, Di Fiore A (2012). A metagenomic study of primate insect diet diversity. Am J Primatol.

[70] Lesnik JJ (2017). Not just a fallback food: global patterns of insect consumption related to geography, not agriculture. Am J Hum Biol.

[71] Cerling TE, Wynn JG, Andanje SA, Bird MI, Korir DK, Levin NE (2011). Woody cover and hominin environments in the past 6 million years. Nature..

[72] Sponheimer M, Alemseged Z, Cerling TE, Grine FE, Kimbel WH, Leakey MG (2013). Isotopic evidence of early hominin diets. Proc Natl Acad Sci.

[73] Ungar PS, Sponheimer M (2011). The diets of early hominins. Science (80- ).

[74] Kourimska L, Adamkova A (2016). Nutritional and sensory quality of edible insects. NFS J.

[75] Rahman NA, Parks DH, Willner DL, Engelbrekston AL, Goffredi SK, Warnecke F (2015). A molecular survey of Australian and north American termite genera indicates that vertical inheritance is the primary force shaping termite gut microbiomes. Microbiome..

[76] Sheppe W (1970). Invertebrate predation on termites of the African savanna. Insect Soc.

[77] Rajakumar R, San Mauro D, Dijkstra MB, Huang MH, Wheeler DE, Hiou-Tim F (2012). Ancestral Developmental Potential Facilitates Parallel Evolution in Ants. Science (80- ).

[78] Marietou A. Nitrate reduction i nsulfate-reducing bacteria. FEMS Microbiol Lett. 2016;363:15. 10.1093/femsle/fnw155 27364687

[79] Müller A, Evans J, Payne CLR, Roberts R (2016). Entomophagy and power. J Insects as Food Feed.

[80] Anklin-Mühlemann R, Bignell DE, Veivers PC, Leuthold RH, Slaytor M (1995). Morphological, microbiological and biochemical studies of the gut flora in the fungus-growing termite Macrotermes subhyalinus. J Insect Physiol.

[81] Arshad MA, Schnitzer M (1987). The chemistry of a termite fungus comb. Plant Soil.

[82] Delsuc F, Metcalf J, Parfrey LW, Song SJ, Gonzalez A, Knight R (2014). Convergence of gut microbiomes in myrmecophagous mammals. Mol Ecol.

[83] Delgado-Baquerizo M, Oliverio AM, Brewer TE, Benavent-González A, Eldridge DJ, Bardgett RD (2018). A global atlas of the dominant bacteria found in soil. Science (80- ).

[84] Caporaso GJ, Lauber CL, Walters WA, Berg-Lyons D, Huntley J, Fierer N (2012). Ultra-high-throughput microbial community analysis on the Illumina HiSeq and MiSeq platforms. ISME J.

[85] Zhang J, Kobert K, Flouri T, Stamatakis A (2014). PEAR: a fast and accurate Illumina paired-end reAd mergeR. Bioinformatics..

[86] Edgar RC, Flyvbjerg H (2015). Error filtering, pair assembly and error correction for next-generation sequencing reads. Bioinformatics..

[87] Edgar RC (2010). Search and clustering orders of magnitude faster than BLAST. Bioinformatics..

[88] Caporaso JG, Kuczynski J, Stombaugh J, Bittinger J, Bushman FD, Costello EK (2010). QIIME allows analysis of high-throughput community sequencing data. Nat Methods.

[89] Edgar RC (2013). UPARSE: highly accurate OTU sequences from microbial amplicon reads. Nat Methods.

[90] Edgar RC (2004). MUSCLE: multiple sequence alignment with high accuracy and high throughput. Nucleic Acids Res.

[91] Price MN, Dehal PS, Arkin AP (2009). FastTree: computing large minimum evolution trees with profiles instead of a distance matrix. Mol Biol Evol.

[92] Faith DP (1992). Conservation evaluation and phylogenetic diversity. Biol Conserv.

[93] Lozupone C, Knight R (2005). UniFrac: a new phylogenetic method for comparing microbial communities. Appl Environ Microbiol.

[94] Andrew S (2010). FastQC: a quality control tool for high throughput sequence data.

[95] Martin M (2011). Cutadapt removes adapter sequences from high-throughput sequencing reads. EMBnet.J.

[96] R Core Team. R: A Language and Environment for Statistical Computing. Vienna: R Foundation for Statistical Computing; 2017. https://www.R-project.org/

[97] Oksanen J, Blanchet FG, Kindt R, Legendre P, Minchin PR, O’Hara RB (2013). Vegan: Community Ecology Package.

[98] Culhane AC, Thioulouse J, Perrière G, Higgins DG (2005). MADE4: an R package for multivariate analysis of gene expression data. Bioinformatics..

[99] Wickham H (2016). Flexibly reshape data: a reboot of the reshape package.

[100] Roberts DW (2016). Ordination and multivariate analysis for ecology.

[101] Ding B, Gentleman R, Carey V (2017). bioDist: Different distance measures.

[102] Shannon P, Markiel A, Ozier O, Baliga NS, Wang JT, Ramage D (2003). Cytoscape: a software environment for integrated models of biomolecular interaction networks. Genome Res.

[103] Katoh K, Misawa K, Kuma K, Miyata T (2002). MAFFT: a novel method for rapid multiple sequence alignment based on fast Fourier transform. Nucleic Acids Res.

[104] Kumar S, Stecher G, Tamura K (2016). MEGA7: molecular evolutionary genetics analysis version 7.0 for bigger datasets. Mol Biol Evol.

