# Supplementary material for: Taxonomic features and comparisons of the gut microbiome from two edible fungus-farming termites (Macrotermes falciger; M. natalensis) harvested in the Vhembe district of Limpopo, South Africa
Source: BMC Microbiol. 2019 Jul 17;19:164. doi: 10.1186/s12866-019-1540-5 (PMC6637627; doi:10.1186/s12866-019-1540-5)
Supplement: Supplementary file 3 — Figure S1. Termite dissection and inventory images. The dissection space and sample documentation shows the set up for sterile sample handling as well as comparisons of termites and examples of a dissected gut. (PDF 2672 kb) [file 12866_2019_1540_MOESM3_ESM.pdf]

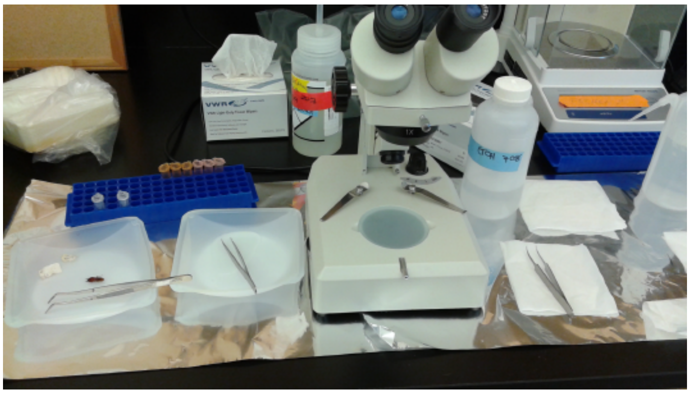

Dissection work station

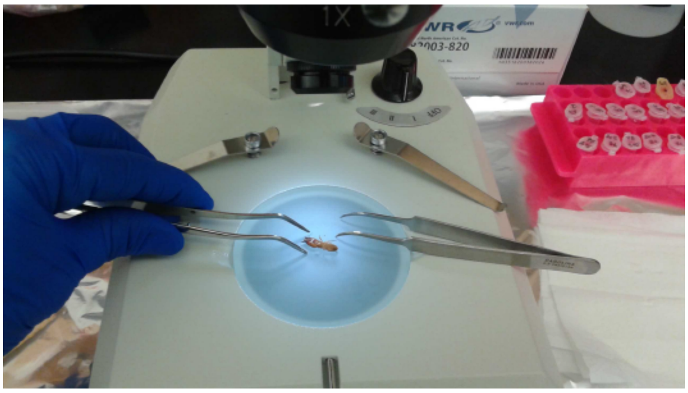

Dissection orientation

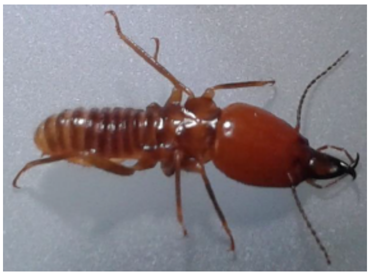

M. falciger minor soldier

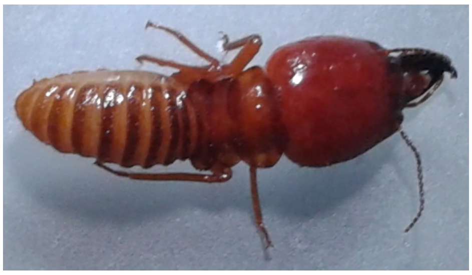

M. falciger major soldier

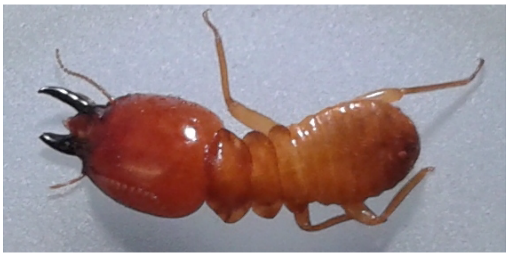

M. natalensis soldier

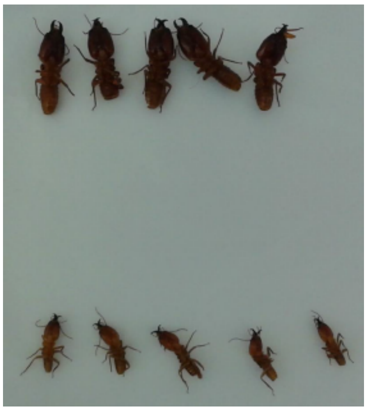

M. falciger major and minor soldiers

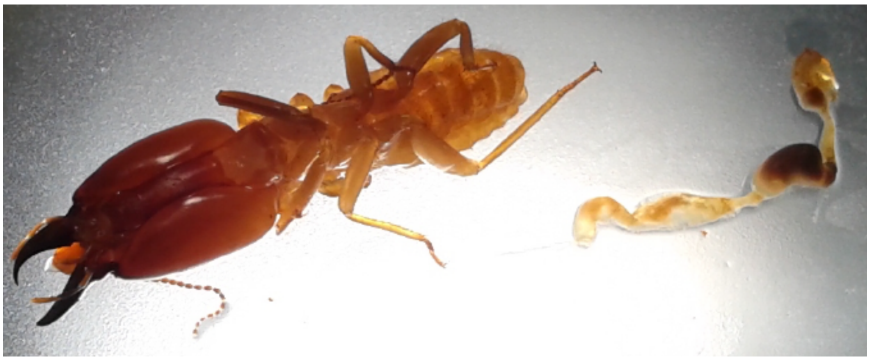

M. falciger dissected soldier

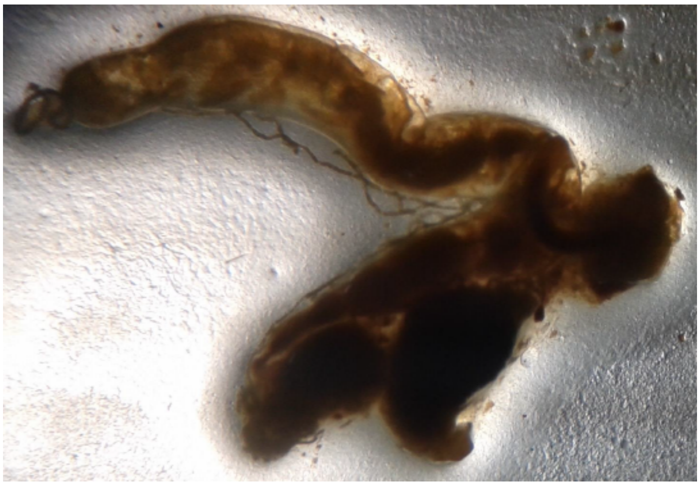

Dissected gut under low magnification

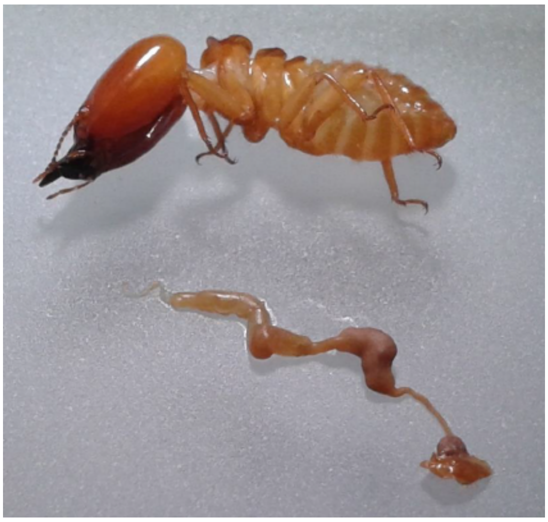

M. natalensis dissected soldier
