# Supplementary figures and images for: Taxonomic features and comparisons of the gut microbiome from two edible fungus-farming termites (Macrotermes falciger; M. natalensis) harvested in the Vhembe district of Limpopo, South Africa
Source: BMC Microbiol. 2019 Jul 17;19:164. doi: 10.1186/s12866-019-1540-5 (PMC6637627; doi:10.1186/s12866-019-1540-5)

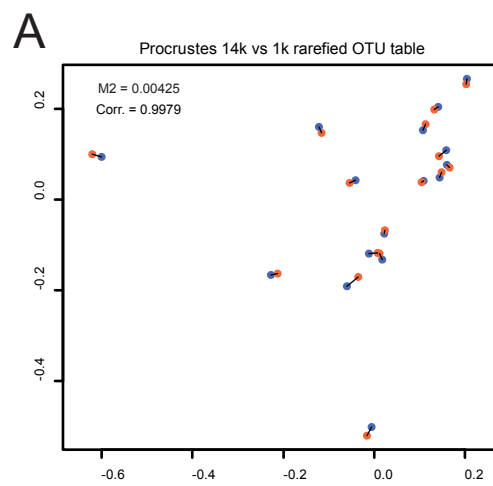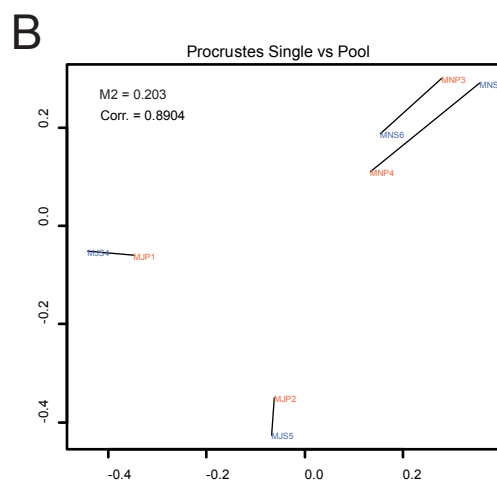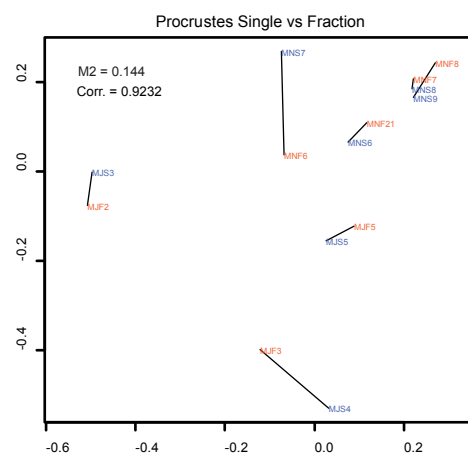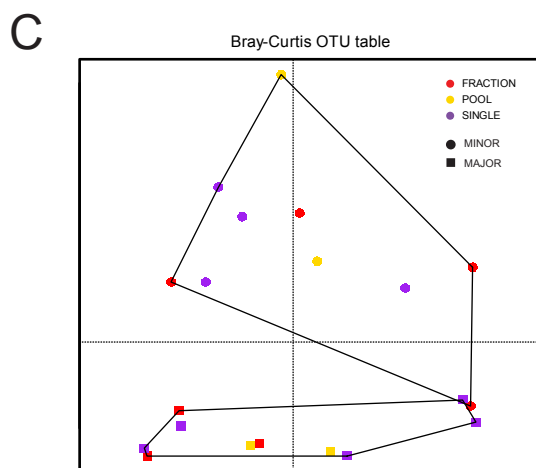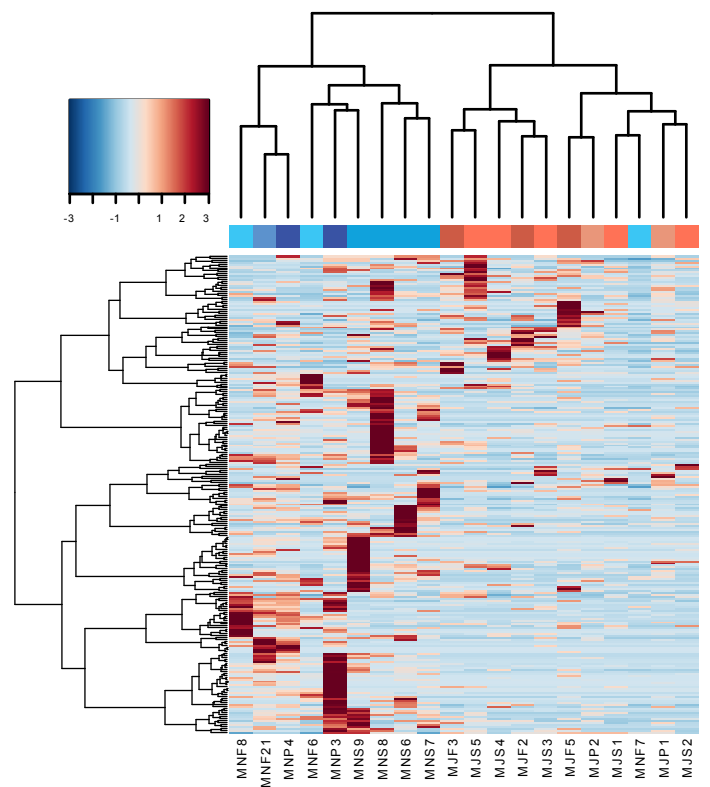

Supplement: Supplementary file 4 — Figure S2. Ecology verification with Run2 samples. (A) Procrustes rotation of 14 k and 1 k rarefied OTU table. (B) Procrustes rotation of single versus pooled and single versus fractioned samples. (C) Bray-Curtis ordination and heatplot of all Run2 samples shows no apparent clustering by extraction type. (PDF 518 kb) [file 12866_2019_1540_MOESM4_ESM.pdf]

A

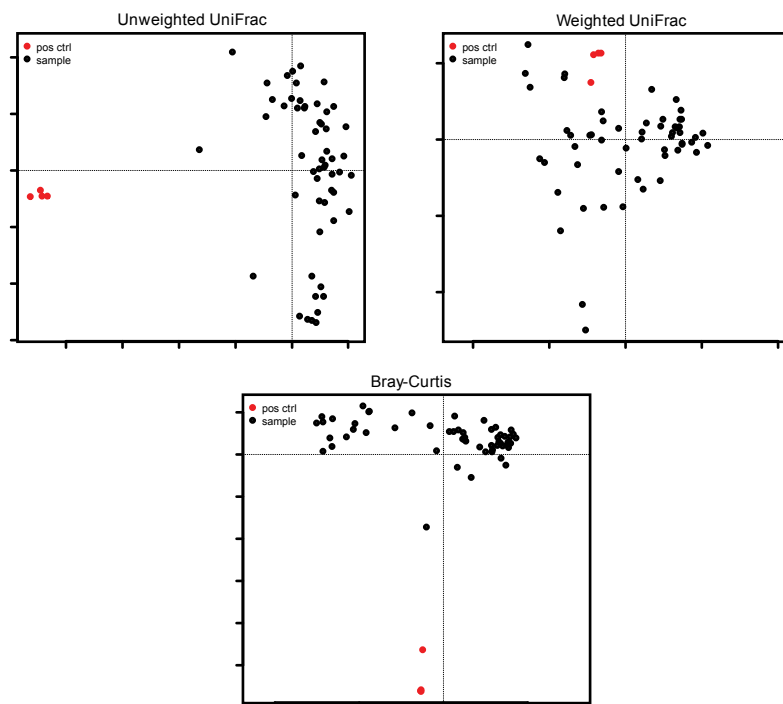

B

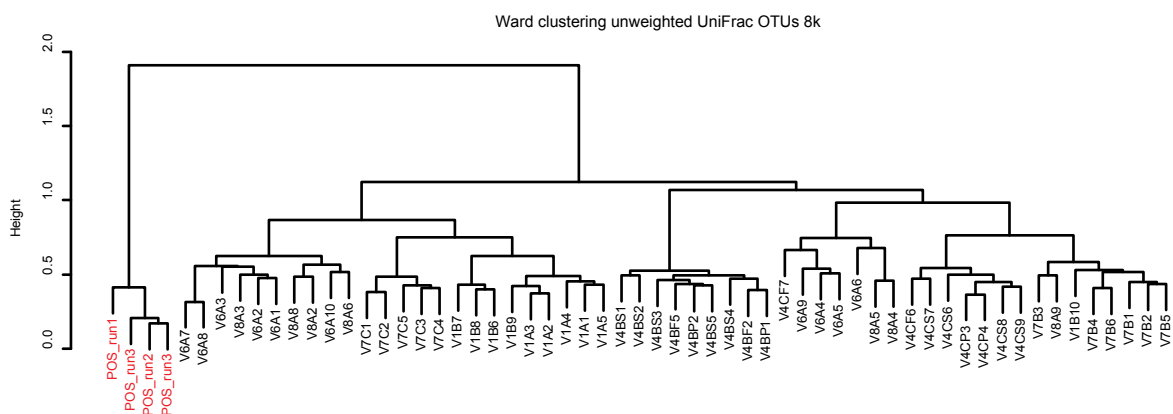

C

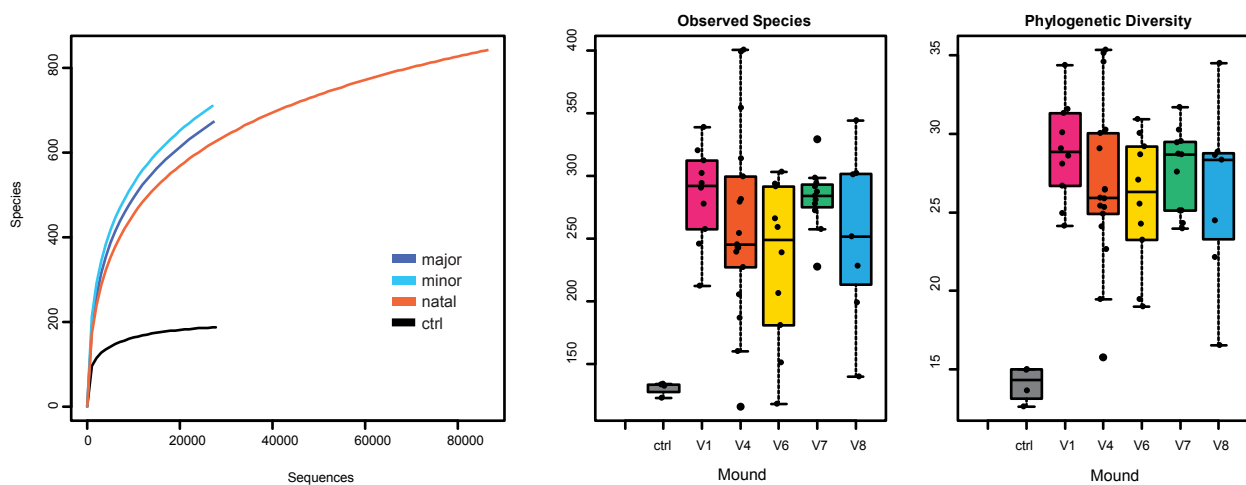

Supplement: Supplementary file 6 — Figure S4. Batch analysis using control samples. (A) Positive gut ecology controls cluster together. (B) Ward clustering confirms ordination. (C) Additional diversity characterization from rarefaction curves and diversity metrics shown by termite mound. (PDF 388 kb) [file 12866_2019_1540_MOESM6_ESM.pdf]

A

## Phylum level (L2)

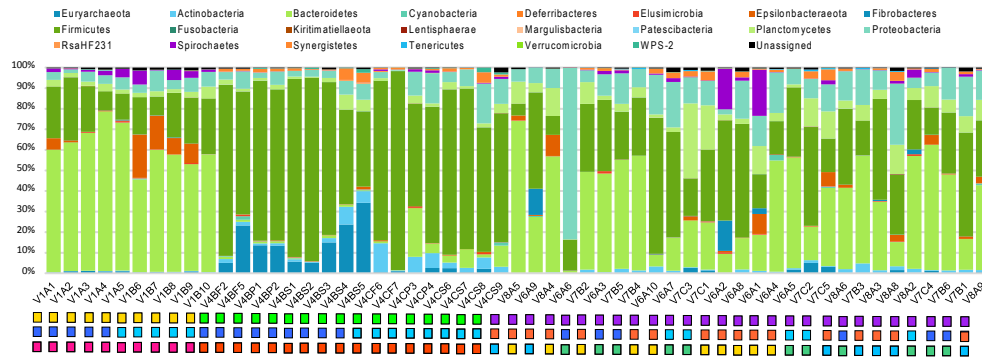

## Genus level (L6)

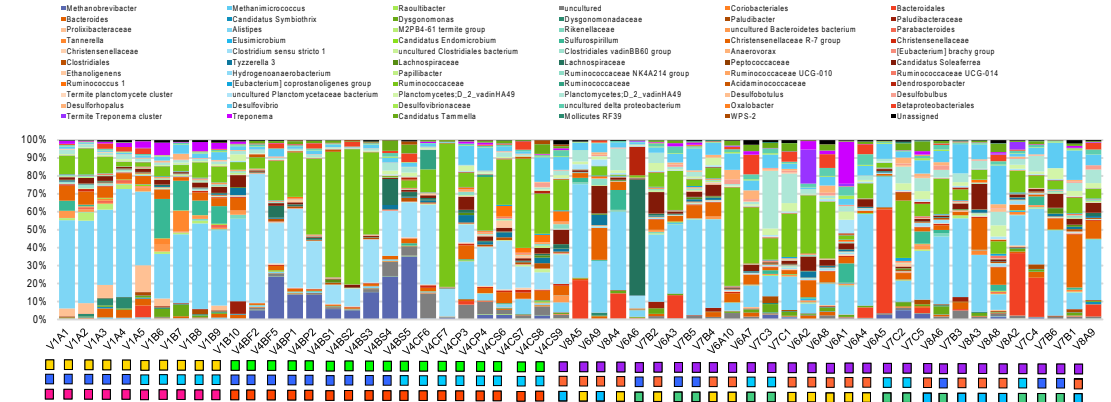

B

## Reference Sequences Genus level

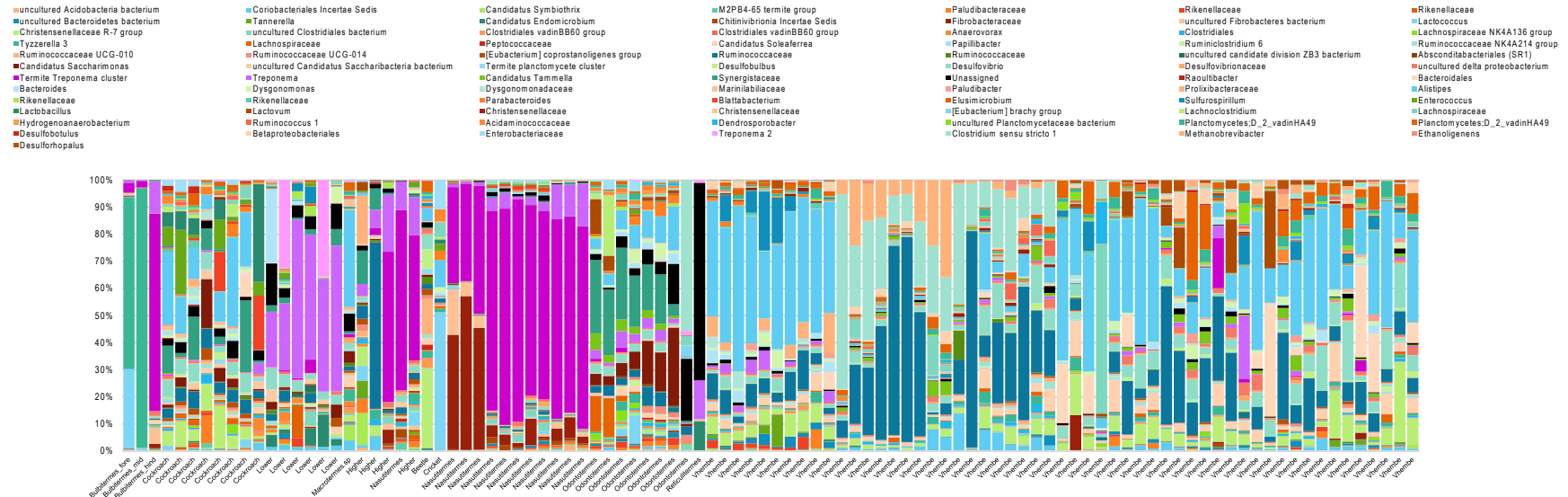

Supplement: Supplementary file 7 — Figure S5. Phylum and genus level taxonomy summaries. (A) Taxonomy summaries at Phylum and Genus level for in-house Vhembe termite data, with color key indicating the run, termite type, and mound, respectively (color key as in Fig. 1). (B) Taxonomy summaries for reference sequence data and in-house data. (PDF 1357 kb) [file 12866_2019_1540_MOESM7_ESM.pdf]

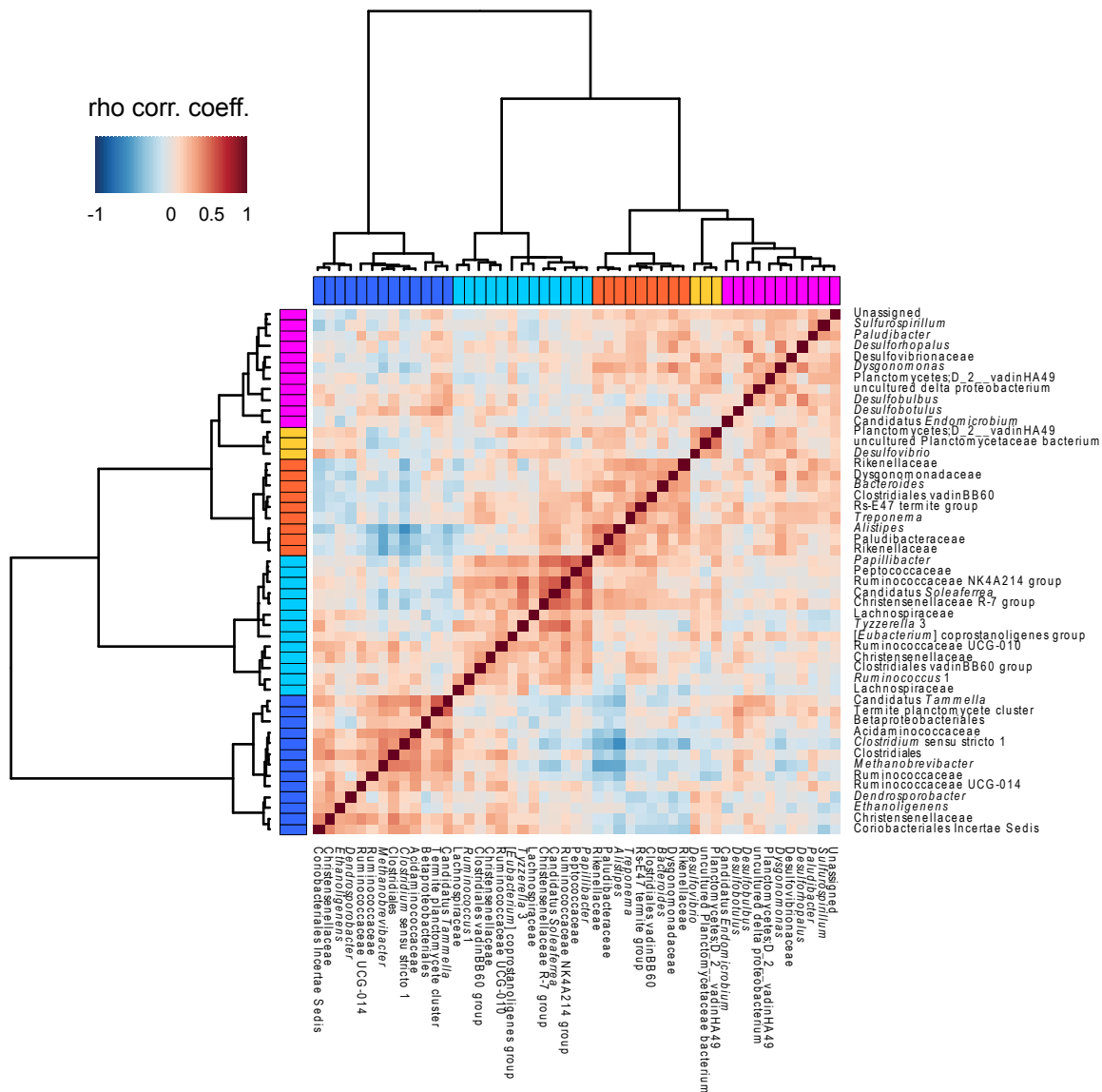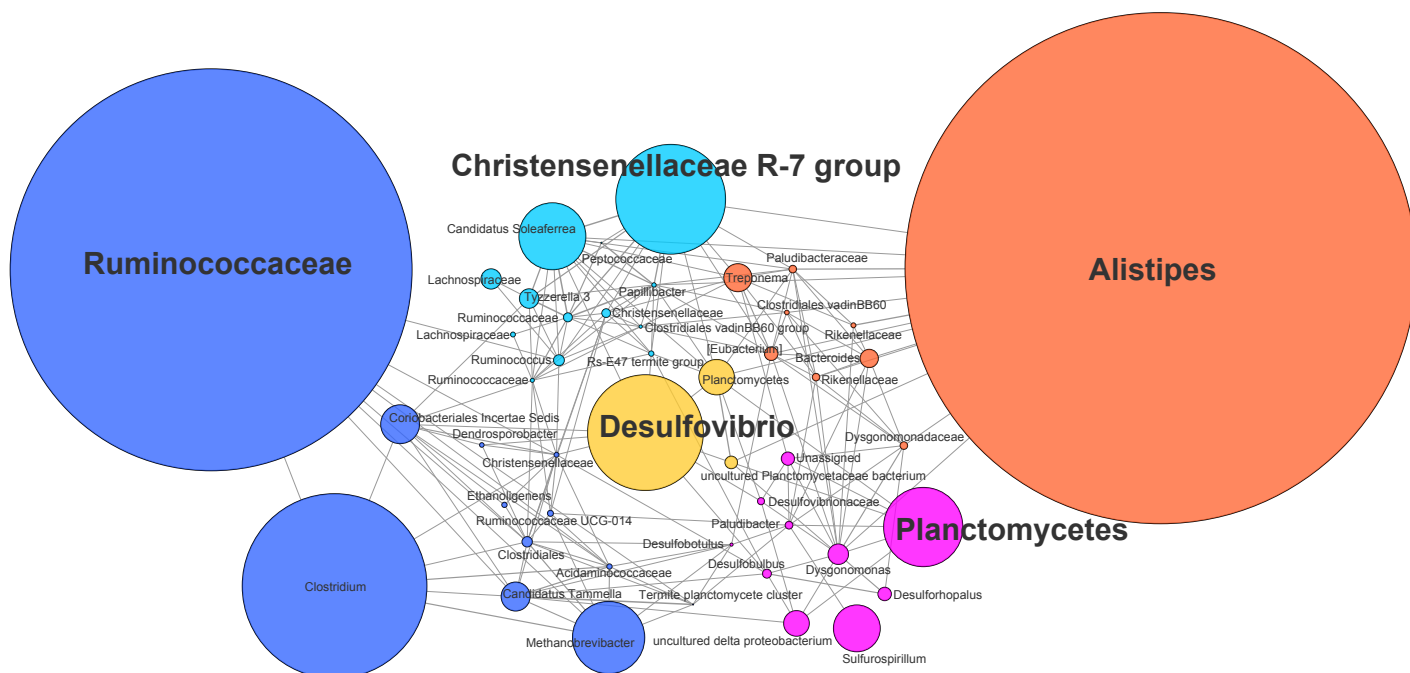

Supplement: Supplementary file 8 — Figure S6. Co-abundance group clustering and network. Correlation heatplot of filtered taxa for in-house samples made with Kendall correlation of abundance and clustered using Ward’s method with Spearman distance. Co-abundance groups were imported into Cytoscape software to visualize the network. Nodes are sized by mean taxonomic abundance and edge widths indicate correlation coefficient. (PDF 494 kb) [file 12866_2019_1540_MOESM8_ESM.pdf]

Reference Sequences NMDS

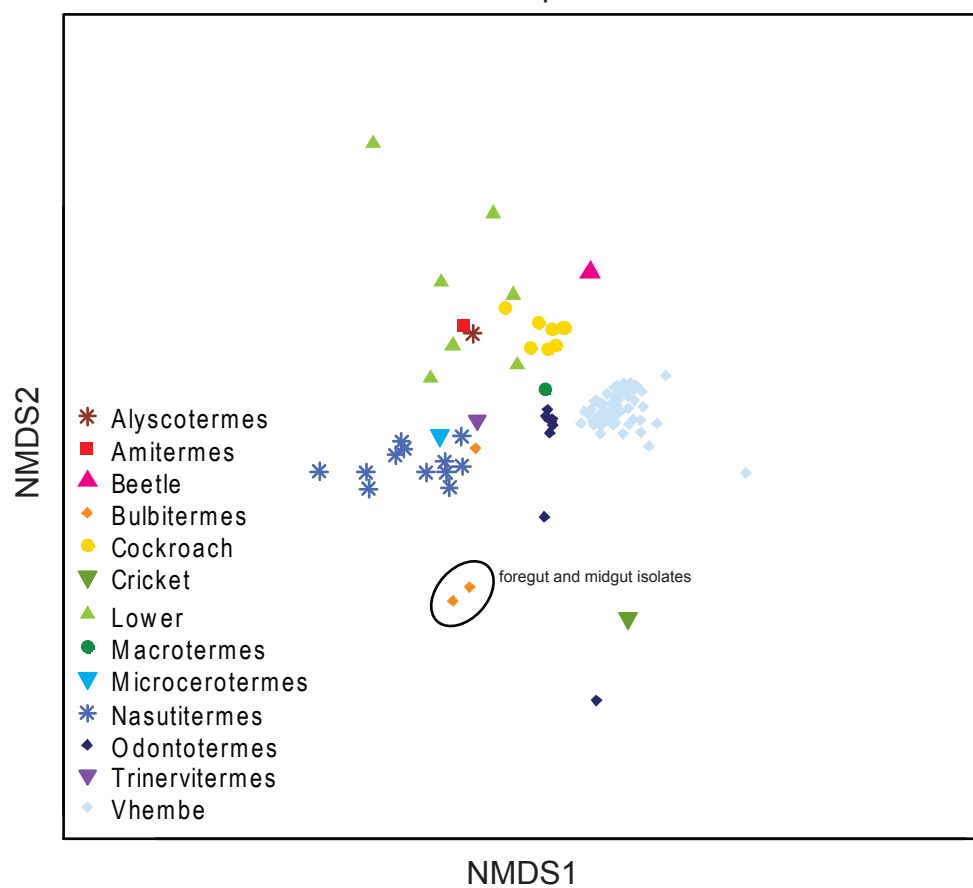

Shepard Plot

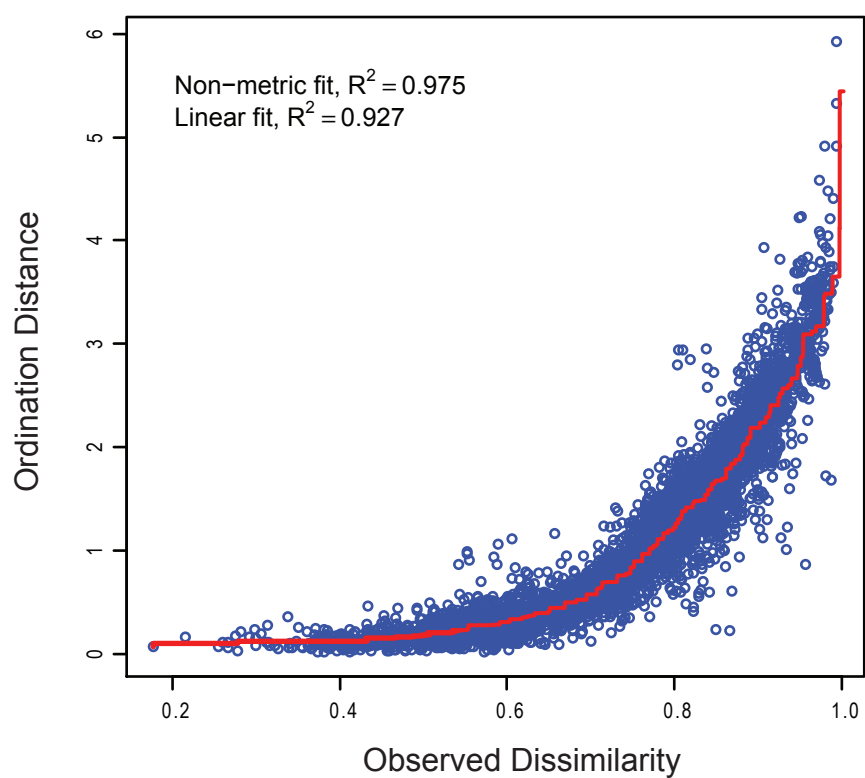

Supplement: Supplementary file 9 — Figure S7. Reference data meta-analysis NMDS. NMDS plot showing stress value and Shepard plot indicating fit. (PDF 849 kb) [file 12866_2019_1540_MOESM9_ESM.pdf]

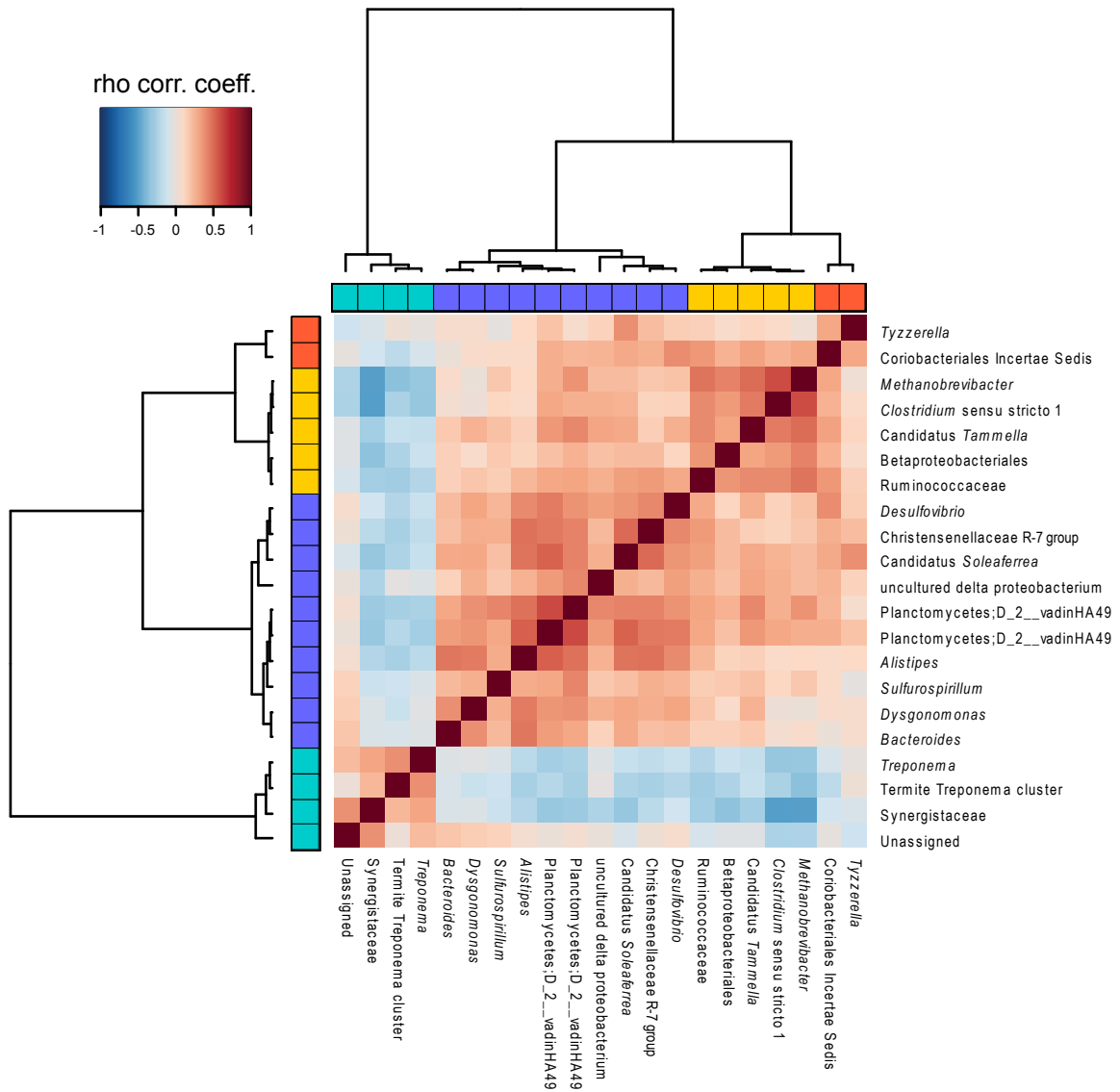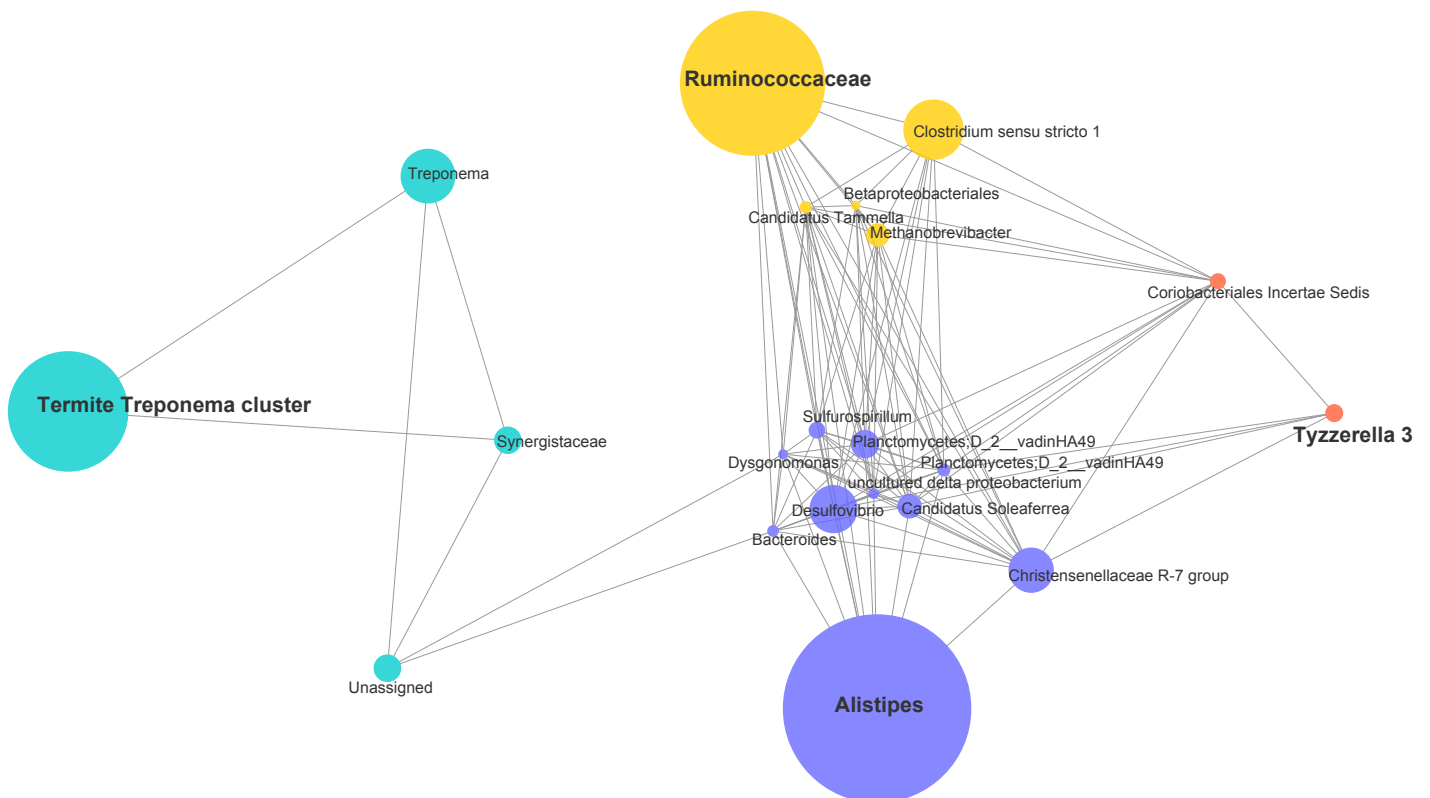

Supplement: Supplementary file 10 — Figure S8. Co-abundance groups for reference meta-data analysis. Three co-abundance groups were found using the same procedure, and visualized in the network. (PDF 316 kb) [file 12866_2019_1540_MOESM10_ESM.pdf]

## Scarab beetle larvae (*Pachnoda ephippiata*)

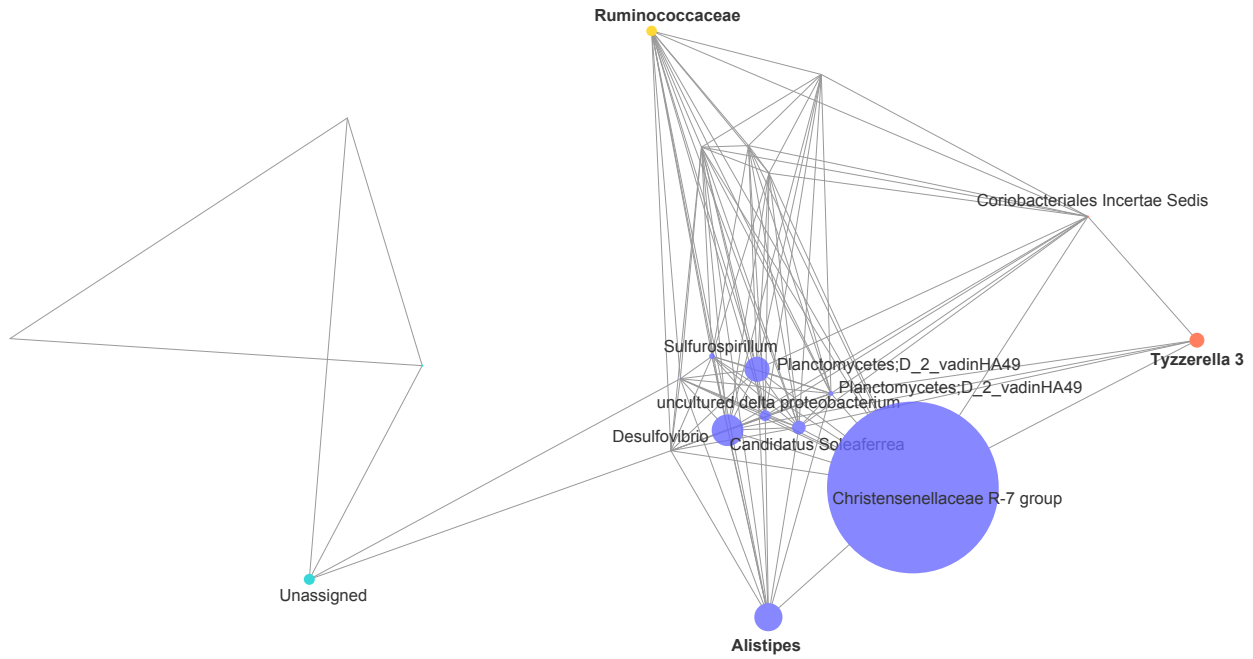

## Field cricket (*Gryllus assimilis*)

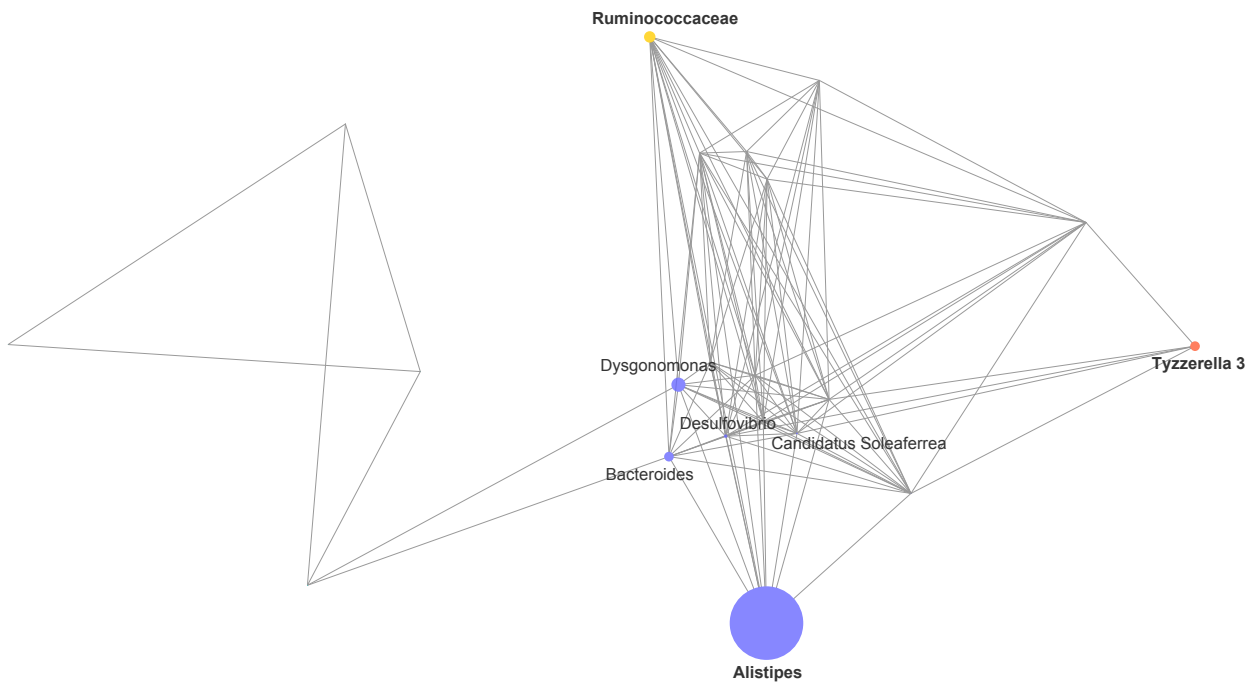

Supplement: Supplementary file 11 — Figure S9. CAGs for other invertebrate data. Scarab beetle larvae CAG best resembles termite fungus farmers and cockroach CAG profiles. (PDF 213 kb) [file 12866_2019_1540_MOESM11_ESM.pdf]

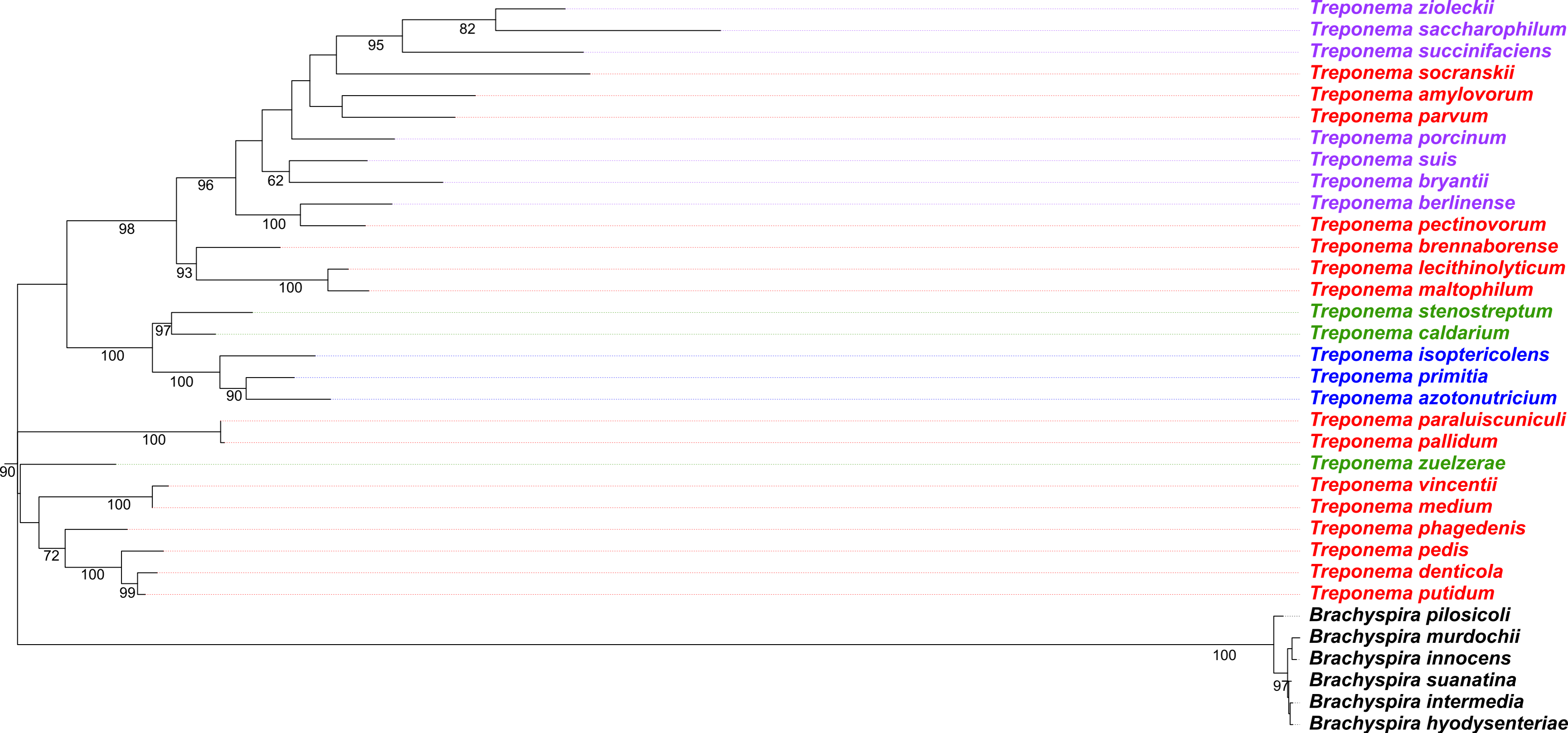

Supplement: Supplementary file 12 — Figure S10. Spirochaetes maximum likelihood tree of full 16S rRNA gene sequences of reference taxa from NCBI. Full length 16S rRNA gene sequences of reference Spirochaetes were first aligned and analyzed to view the sequence relationships using more sites. The percentage of trees in which the associated taxa clustered together is shown next to the branches. Branch labels are color-coded based on strain association to source and pathogenicity: red - pathogenic Treponema; purple - non-pathogenic Treponema; green - environmental Treponema; blue - Treponema species associated with termite gut. (PDF 197 kb) [file 12866_2019_1540_MOESM12_ESM.pdf]
