# Supplementary material for: Taxonomic features and comparisons of the gut microbiome from two edible fungus-farming termites (Macrotermes falciger; M. natalensis) harvested in the Vhembe district of Limpopo, South Africa
Source: BMC Microbiol. 2019 Jul 17;19:164. doi: 10.1186/s12866-019-1540-5 (PMC6637627; doi:10.1186/s12866-019-1540-5)
Supplement: Supplementary file 5 — Figure S3. Metadata correlation of Run2 dissection and extraction data and taxonomic abundance. Dissection and extraction variables do not indicate biases in alpha-diversity metrics based on gut mass, extraction concentration, and qPCR Cq values. Taxonomic bias is also not apparent. (PDF 421 kb) [file 12866_2019_1540_MOESM5_ESM.pdf]

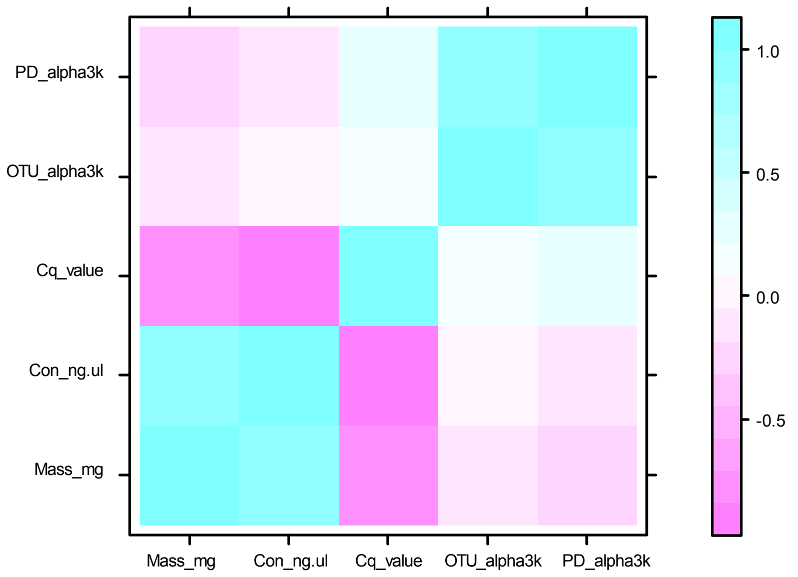

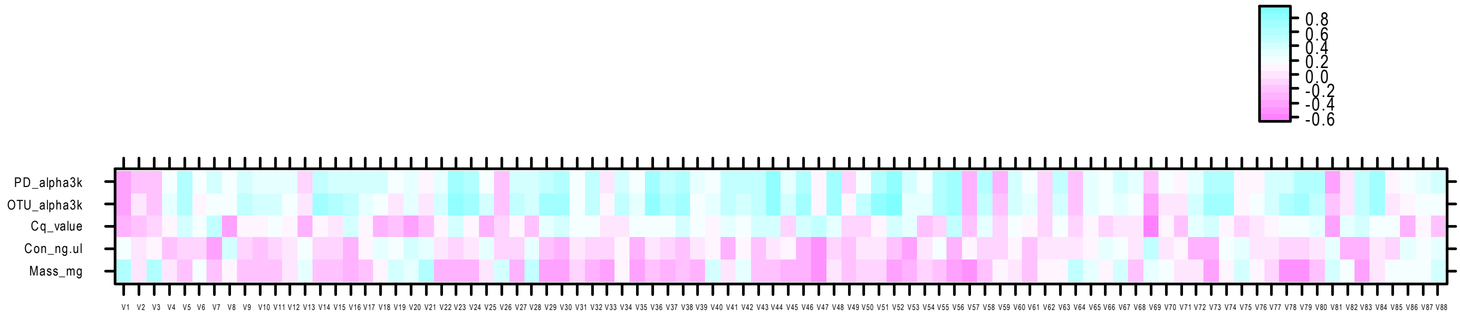

V1 Methanobacteriales;D_4__Methanobacteriaceae;D_5__Methanobrevibacter
V2 Bifidobacteriales;D_4__Bifidobacteriaceae;D_5__Alloscardovia
V3 Micrococcales;D_4__Cellulomonadaceae;D_5__Cellulomonas
V4 Coriobacteriales;D_4__Coriobacteriales Incertae Sedis;D_5__Raoultibacter
V5 Coriobacteriales;D_4__Coriobacteriales Incertae Sedis;D_5__uncultured
V6 Coriobacteriales;D_4__uncultured;D_5__uncultured bacterium
V7 OPB41;Other;Other
V8 Bacteroidales;D_4__3M1PL1-52 termite group;D_5__uncultured bacterium
V9 Bacteroidales;D_4__Bacteroidaceae;D_5__Bacteroides
V10 Bacteroidales;D_4__Dysgonomonadaceae;D_5__Candidatus Symbiothrix
V11 Bacteroidales;D_4__Dysgonomonadaceae;D_5__Dysgonomonas
V12 Bacteroidales;D_4__Paludibacteraceae;D_5__Paludibacter
V13 Bacteroidales;D_4__Prolixibacteraceae;D_5__uncultured
V14 Bacteroidales;D_4__Rikenellaceae;D_5__Alistipes
V15 Bacteroidales;D_4__Rikenellaceae;D_5__uncultured
V16 Bacteroidales;D_4__Rs-E47 termite group;D_5__uncultured Bacteroidetes bacterium
V17 Bacteroidales;D_4__Tannerellaceae;D_5__Tannerella
V18 Gastranaerophilales;D_4__uncultured bacterium;D_5__uncultured bacterium
V19 Gastranaerophilales;Other;Other
V20 Endomicrobiales;D_4__Endomicrobiaceae;D_5__Candidatus Endomicrobium
V21 Campylobacterales;D_4__Sulfurospirillaceae;D_5__Sulfurospirillum
V22 Lactobacillales;D_4__Streptococcaceae;D_5__Lactococcus
V23 Clostridiales;D_4__Christensenellaceae;D_5__Christensenellaceae R-7 group
V24 Clostridiales;D_4__Christensenellaceae;D_5__uncultured
V25 Clostridiales;D_4__Christensenellaceae;Other
V26 Clostridiales;D_4__Clostridiaceae 1;D_5__Clostridium sensu stricto 1
V27 Clostridiales;D_4__Clostridiales vadinBB60 group;D_5__uncultured Clostridiales bacterium
V28 Clostridiales;D_4__Clostridiales vadinBB60 group;D_5__uncultured bacterium
V29 Clostridiales;D_4__Clostridiales vadinBB60 group;Other
V30 Clostridiales;D_4__Family XIII;D_5__Anaerovorax
V31 Clostridiales;D_4__Family XIII;D_5__[Eubacterium] brachy group
V32 Clostridiales;D_4__Family XIII;D_5__uncultured
V33 Clostridiales;D_4__Gracilibacteraceae;D_5__Lutispora
V34 Clostridiales;D_4__Lachnospiraceae;D_5__Anaerocolumna
V35 Clostridiales;D_4__Lachnospiraceae;D_5__Lachnoclostridium 12
V36 Clostridiales;D_4__Lachnospiraceae;D_5__Lachnospira
V37 Clostridiales;D_4__Lachnospiraceae;D_5__Roseburia
V38 Clostridiales;D_4__Lachnospiraceae;D_5__Tyzzerella 3
V39 Clostridiales;D_4__Lachnospiraceae;D_5__uncultured
V40 Clostridiales;D_4__Lachnospiraceae;Other
V41 Clostridiales;D_4__Peptococcaceae;D_5__Desulfosporosinus
V42 Clostridiales;D_4__Peptococcaceae;D_5__uncultured
V43 Clostridiales;D_4__Ruminococcaceae;D_5__Anaerotruncus
V44 Clostridiales;D_4__Ruminococcaceae;D_5__Candidatus Soleaferrea
V45 Clostridiales;D_4__Ruminococcaceae;D_5__Ethanoligenens
V46 Clostridiales;D_4__Ruminococcaceae;D_5__Hydrogenoanaerobacterium
V47 Clostridiales;D_4__Ruminococcaceae;D_5__Oscillibacter
V48 Clostridiales;D_4__Ruminococcaceae;D_5__Papillibacter
V49 Clostridiales;D_4__Ruminococcaceae;D_5__Ruminiclostridium 5
V50 Clostridiales;D_4__Ruminococcaceae;D_5__Ruminiclostridium 6
V51 Clostridiales;D_4__Ruminococcaceae;D_5__Ruminococcaceae NK4A214 group
V52 Clostridiales;D_4__Ruminococcaceae;D_5__Ruminococcaceae UCG-010
V53 Clostridiales;D_4__Ruminococcaceae;D_5__Ruminococcaceae UCG-013
V54 Clostridiales;D_4__Ruminococcaceae;D_5__Ruminococcaceae UCG-014
V55 Clostridiales;D_4__Ruminococcaceae;D_5__Ruminococcus 1
V56 Clostridiales;D_4__Ruminococcaceae;D_5__[Eubacterium] coprostanoligenes group
V57 Clostridiales;D_4__Ruminococcaceae;D_5__uncultured
V58 Clostridiales;D_4__Ruminococcaceae;Other
V59 Clostridiales;D_4__Syntrophomonadaceae;D_5__Syntrophomonas
V60 Clostridiales;Other;Other
V61 Erysipelotrichales;D_4__Erysipelotrichaceae;D_5__Breznakia
V62 Selenomonadales;D_4__Acidaminococcaceae;D_5__uncultured
V63 Selenomonadales;D_4__Veillonellaceae;D_5__Dendrosporobacter
V64 Pirellulales;D_4__Pirellulaceae;D_5__Termite planctomycete cluster
V65 D_2__vadinHA49;D_3__uncultured bacterium;D_4__uncultured bacterium;D_5__uncultured bacterium
V66 uncultured planctomycete;D_4__uncultured planctomycete;D_5__uncultured planctomycete
V67 D_2__vadinHA49;Other;Other;Other
V68 Rhodospirillales;D_4__uncultured;D_5__uncultured bacterium
V69 Rhodospirillales;D_4__uncultured;Other
V70 Desulfarculales;D_4__Desulfarculaceae;D_5__uncultured
V71 Desulfobacterales;D_4__Desulfobacteraceae;D_5__Desulfobotulus
V72 Desulfobacterales;D_4__Desulfobulbaceae;D_5__Desulfobulbus
V73 Desulfobacterales;D_4__Desulfobulbaceae;D_5__Desulforhopalus
V74 Desulfovibrionales;D_4__Desulfovibrionaceae;D_5__Desulfovibrio
V75 Desulfovibrionales;D_4__Desulfovibrionaceae;D_5__uncultured
V76 Rs-K70 termite group;D_4__uncultured delta proteobacterium;D_5__uncultured delta proteobacterium
V77 Betaproteobacteriales;D_4__Burkholderiaceae;D_5__Oxalobacter
V78 Betaproteobacteriales;D_4__Nitrosomonadaceae;D_5__Nitrosomonas
V79 Betaproteobacteriales;D_4__Rhodocyclaceae;D_5__Dechloromonas
V80 Betaproteobacteriales;D_4__Rhodocyclaceae;Other
V81 Betaproteobacteriales;Other;Other
V82 D_1__RsaHF231;D_2__uncultured bacterium;D_3__uncultured bacterium;D_4__uncultured bacterium;D_5__uncultured bacterium
V83 Spirochaetales;D_4__Spirochaetaceae;D_5__Termite Treponema cluster
V84 Spirochaetales;D_4__Spirochaetaceae;D_5__Treponema
V85 Synergistales;D_4__Synergistaceae;D_5__Candidatus Tammella
V86 Mollicutes RF39;D_4__uncultured bacterium;D_5__uncultured bacterium
V87 D_1__WPS-2;D_2__uncultured bacterium;D_3__uncultured bacterium;D_4__uncultured bacterium;D_5__uncultured bacterium
V88 Unassigned;Other;Other;Other;Other;Other
